# Simple, scalable mechanosynthesis of metal–organic frameworks using liquid-assisted resonant acoustic mixing (LA-RAM)†

**DOI:** 10.1039/d0sc00333f

**Published:** 2020-02-27

**Authors:** Hatem M. Titi, Jean-Louis Do, Ashlee J. Howarth, Karthik Nagapudi, Tomislav Friščić

**Affiliations:** Department of Chemistry, McGill University 801 Sherbrooke St. West Montreal QC H3A 0B8 Canada tomislav.friscic@mcgill.ca; Department of Chemistry and Biochemistry, Concordia University Montreal QC Canada; Genentech One Dna Way South San Francisco CA 94080 USA

## Abstract

We present a rapid and readily scalable methodology for the mechanosynthesis of diverse metal–organic frameworks (MOFs) in the absence of milling media typically required for other types of mechanochemical syntheses. We demonstrate the use of liquid-assisted resonant acoustic mixing (LA-RAM) methodology for the synthesis of three- and two-dimensional MOFs based on Zn(ii), Co(ii) and Cu(ii), including a mixed ligand system. Importantly, the LA-RAM approach also allowed the synthesis of the ZIF-L framework that has never been previously obtained in a mechanochemical environment, as well as its Co(ii) analogue. Straightforward scale-up from milligrams to at least 25 grams is demonstrated using the metastable framework ZIF-L as the model.

## Abstract 2

We present a rapid and readily scalable methodology for the mechanosynthesis of diverse metal–organic frameworks (MOFs) in the absence of milling media typically required for other types of mechanochemical syntheses.

## Graphical abstract

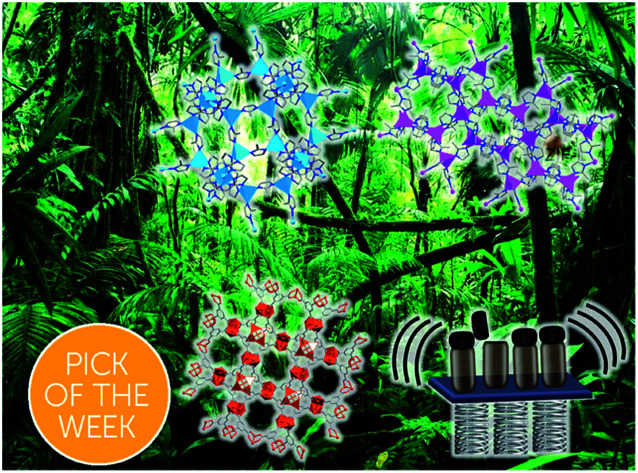

## Introduction

**Fig. 1:**
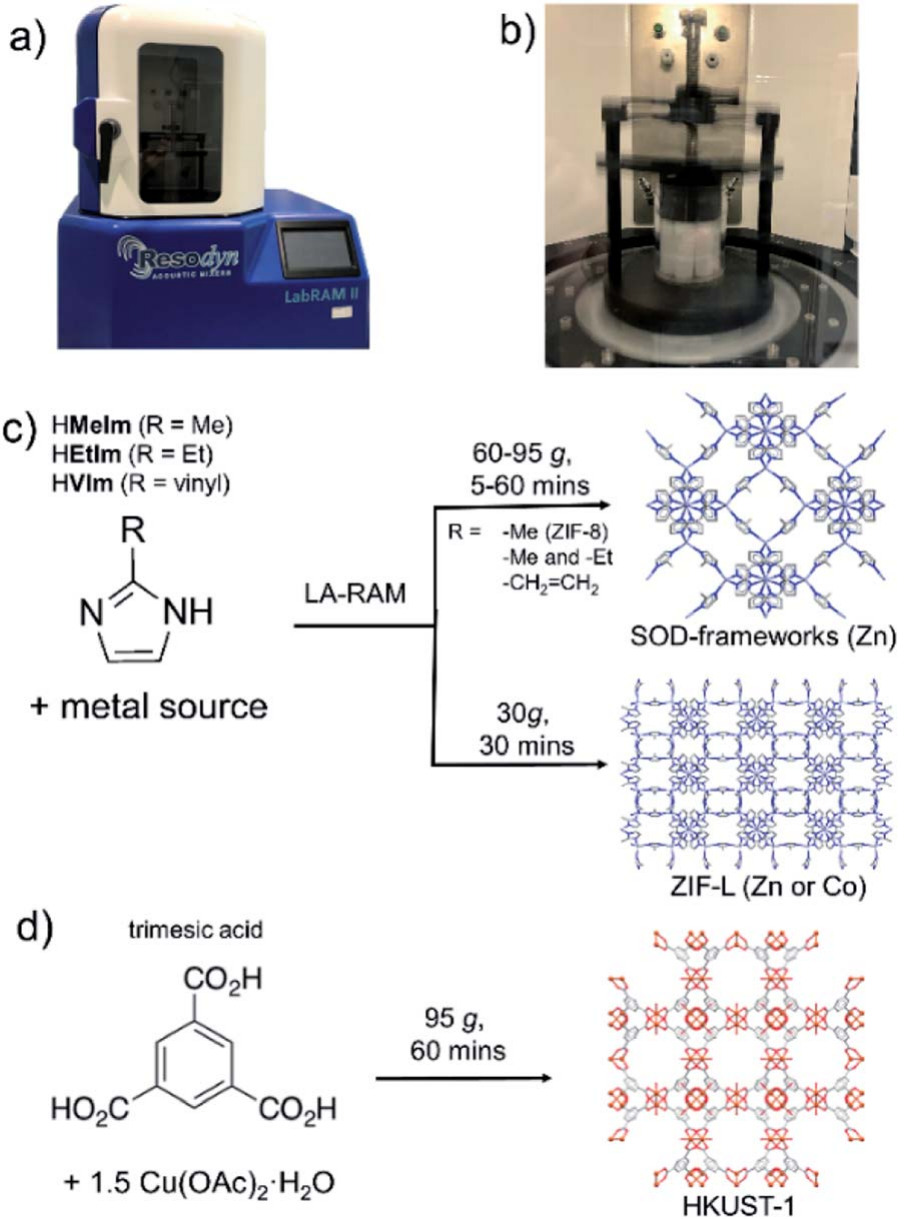
Herein employed LabRam resonant acoustic mixer: (a) exterior and (b) interior shown during operation. Schematic illustration of herein developed MOF syntheses using RAM: (c) ZIF-8, mixed-ligand SOD-Zn(**MeIm**)(**EtIm**) and ZIF-L (Zn and Co variants) and (d) copper-based HKUST-1.

Mechanochemical synthesis,^1^ conducted by milling, grinding or shearing, has over the past decade been deployed in a wide range of processes, from the synthesis of pharmaceutically active ingredients^2^ and cocrystals,^3^ to metal–organic frameworks (MOFs)^4^ and nanoparticle materials.^5^ Rapid, room-temperature reactions in a solvent-free or solvent-limited mechanochemical environment are not only highly attractive in the context of Green Chemistry, but also provide access to new materials, reactions and reaction selectivity that is difficult to achieve in conventional solution-based syntheses.^6,7^ Typically, mechanochemical reactions on the laboratory or manufacturing scale are conducted by using ball milling^8^ or twin screw extrusion.^9^ While these approaches rely on the grinding or shear forces created by the grinding media or rotating screws in the equipment, recent reports have begun investigating mechanochemical reactivity without such components, *e.g.* by ultrasonic^10^ or acoustic^11^ frequency sample agitation (Fig. 1a and b). These two nascent methodologies are highly promising as, in principle, they offer a route for considerable simplification of sample preparation and scaling up. However, they have so far been applied exclusively to cocrystal formation.^12^

As the next step in developing cleaner, simpler and readily scalable synthetic methodologies that do not use bulk solvents, we now demonstrate the application of resonant acoustic mixing (RAM)^11,12^ for the assembly of MOFs. This proof-of-principle study shows how the versatility of RAM can be enhanced by a small amount of a liquid additive, in a process analogous to liquid-assisted grinding (LAG)^13^ previously used to advance the efficiency and scope of ball milling mechanochemistry, to enable the synthesis of two- (2-D) and three-dimensional (3-D) MOFs without bulk solvent or any grinding media. This liquid-assisted resonant acoustic mixing (LA-RAM) methodology is demonstrated for simple, rapid synthesis of imidazolate and carboxylate MOFs based on Zn(ii) or Cu(ii), including a mixed-ligand system, as well as commercially-relevant frameworks ZIF-8 and HKUST-1 (Fig. 1c and d). Importantly, LA-RAM also enabled a simple, rapid route for making the metastable 2-D layered MOF material ZIF-L^13,14^ and its Co(ii) analogue, neither of which have previously been obtained *via* mechanochemistry. Using ZIF-L as a model, the scale-up of LA-RAM synthesis of MOFs from hundreds of milligrams to at least 25 grams is demonstrated.

All described RAM reactions were done using a Resodyn LabRAM II system (Fig. 1a and b),^15^ operating in auto-resonance mode of 60 Hz. In most cases, the reaction mixtures were contained in 8 mL plastic vials, at 200–300 mg scale. Scale-up reactions were conducted on the scale from *ca.* 1.5 grams (for ZIF-8, SOD-Zn(**MeIm**)(**EtIm**) materials) to 25 grams (for ZIF-L) of product. Products were characterized by powder X-ray diffraction (PXRD), thermogravimetric analysis (TGA), Fourier-transform infrared attenuated total reflectance (FTIR-ATR) spectroscopy, N_2_ sorption analysis, solution- and solid-state nuclear magnetic resonance (NMR) spectroscopy, and scanning electron microscopy (SEM). Further details of synthetic procedures and instrumental techniques are given in the ESI.†

## Results and discussion

**Fig. 2:**
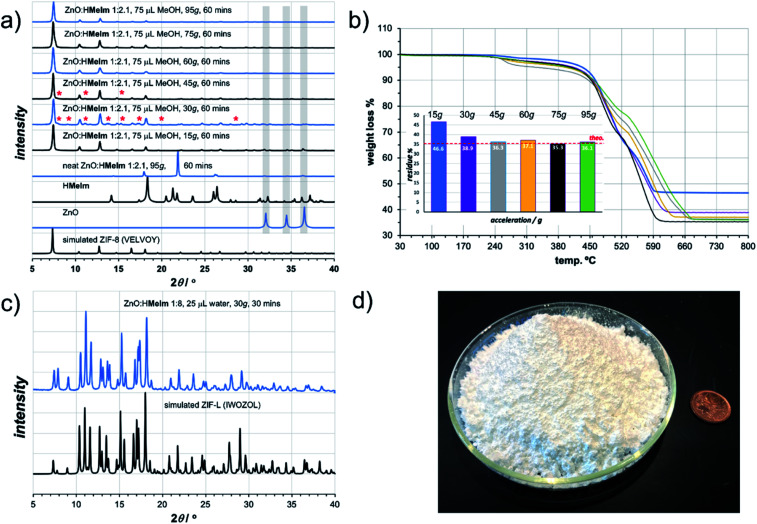
Comparison of selected: (a) PXRD patterns and (b) TGA thermograms for ZIF-8 materials obtained by LA-RAM for 60 minutes in the presence of MeOH (liquid-to-solid ratio *η* ≈ 0.3 μL mg^−1^) and NH_4_NO_3_ (5 mol% with respect to Zn) at different *g* acceleration values. The TGA measurements are reported for samples after washing and activation. The PXRD patterns of products obtained using accelerations of 30*g* and 45*g* reveal the presence of ZIF-L impurity, highlighted by “*”. (c) PXRD pattern for ZIF-L obtained by LA-RAM with water as the additive, compared to the simulated patterns for the reported crystal structure (CSD code IWOZOL) and (d) image of a 25 gram sample of ZIF-L obtained by LAG, compared to a one cent Canadian coin.

As a first attempt of RAM-based MOF mechanosynthesis, we investigated the reaction of ZnO and 2-methylimidazole (H**MeIm**) to form the popular sodalite (SOD) topology Zn(**MeIm**)_2_ framework (ZIF-8, MAF-4).^16–18^ The principal parameters expected to affect a chemical reaction in a resonant acoustic mixer are time and the acceleration experienced by the sample. The acceleration is expressed in *g* units (*g* = 9.81 m s^−2^) and is varied by changing the amplitude of acoustic agitation. It was previously observed that higher *g* values lead to improved mixing and reactivity in cocrystal synthesis.^12^ The maximum achievable acceleration in the herein used Resodyn acoustic mixer was 95*g*. Agitation of a neat mixture of ZnO and H**MeIm** in a 1 : 2.1 stoichiometric ratio (5 mol% excess H**MeIm**) at 95*g* led to no reaction after 60 minutes, as evidenced by the PXRD pattern of the reaction mixture exhibiting only Bragg reflections of solid reactants (Fig. 2a). Similarly, RAM at 95*g* in the presence of a small amount of MeOH (75 μL, corresponding to the liquid-to-solid ratio^19^*η* = 0.30 μL mg^−1^, see ESI†) led to only to trace amounts of ZIF-8 after an hour. The poor reactivity of ZnO and H**MeIm** mixtures upon RAM at 95*g* is very different from that observed in ball milling, where both neat milling and LAG easily lead to incomplete but significant formation of ZIF-8.^20^ The reaction outcome did not change significantly even when the RAM process was performed in the presence of additional milling media, in the form of 10 zirconia (ZrO_2_) balls of 3 mm diameter (see ESI†).

Next, we explored RAM with MeOH (*η* = 0.30 μL mg^−1^), but this time in the presence of a catalytic amount of NH_4_NO_3_ (5 mol% with respect to Zn), mimicking the ion- and liquid-assisted grinding (ILAG) conditions^17^ previously shown to enable quantitative synthesis of ZIF-8 from ZnO by ball milling. Under these conditions, liquid-assisted RAM (LA-RAM) for 1 hour at 95*g* led to complete transformation of ZnO to ZIF-8, without the need for any milling media, as evidenced by PXRD and TGA of the product after washing with MeOH and evacuation (Fig. 2a and b). The sample temperature was measured before and immediately after mixing, revealing only a 0.7 °C temperature increase over 60 minutes, indicating that the reactivity is most likely driven by improved contact and mixing, rather than a bulk heating effect. The LA-RAM synthesis of ZIF-8 was also readily accomplished using acetone, ethanol (EtOH), acetonitrile (CH_3_CN) or *N*,*N*-dimethylformamide (DMF) as liquid additives (*η* ≈ 0.3 μL mg^−1^). The conversion to ZIF-8 was also not significantly affected by varying the amount of liquid phase, with complete conversion of ZnO observed upon LA-RAM in the presence of either 100 μL, 75 μL or even 50 μL of MeOH (see ESI†).

After establishing conditions for production of ZIF-8 at 95*g*, we explored reactivity under milder conditions at 15, 30, 45, 60 and 75*g* (Fig. 2a). As confirmed by PXRD and TGA (Fig. 2b), quantitative conversion of ZnO to ZIF-8 as the sole product was observed at 60*g* and 75*g*, with lower acceleration giving only partial transformation of ZnO after one-hour of RAM, presumably due to poorer mixing.

After optimizing reaction composition and RAM acceleration, we next focused on the reaction time. Analysis of the reaction mixture containing ZnO, H**MeIm**, and catalytic amounts of MeOH and NH_4_NO_3_ after RAM treatment at 95*g* for different time periods revealed almost complete disappearance of ZnO after 5 minutes. However, LA-RAM for 15 minutes or more always provided complete conversion to ZIF-8 (Fig. 2a and b). The resulting product, after washing with MeOH and evacuation provided a Brunauer–Emmett–Teller (BET) surface area of 1200 m^2^ g^−1^ (Table 1, also see ESI†).^21^ Importantly, the reaction was also readily scaled at least 5-fold, providing access to gram quantities of ZIF-8 within only 60 minutes (see ESI†).

**Table 1:** Measured BET surface areas and typical particle sizes (by SEM) for selected samples of microporous MOFs prepared using LA-RAM, after washing and evacuation

MOF	BET area (m^2^ g^−1^)	Particle size (nm)
Zn(**MeIm**)_2_^a^^,^^b^	1200	50–200
Zn(**VIm**)_2_^a^^,^^b^	1110	50–300
Zn(**EtIm**)(**MeIm**)^a^^,^^c^	1140	40–90
HKUST-1^a^^,^^d^	1310	40–90

a All samples prepared at 95*g*.

b Using MeOH as liquid additive.

c Using CH_3_CN as liquid additive.

d Using water as liquid additive.

The ease and simplicity with which ZIF-8 was obtained through LA-RAM in the presence of a protic salt catalyst led us to investigate other ligand systems. Using the same optimized conditions identified for ZIF-8 synthesis, the analogous SOD-topology framework based on 2-vinylimidazole (H**VIm**, Fig. 1c) was obtained in quantitative conversion (see ESI†),^22^ as evidenced by PXRD, and TGA. Nitrogen sorption evaluation of SOD-Zn(**VIm**)_2_ gave a BET surface area of 1110 m^2^ g^−1^, comparable to previous reports (see ESI†).^22^

**Fig. 3:**
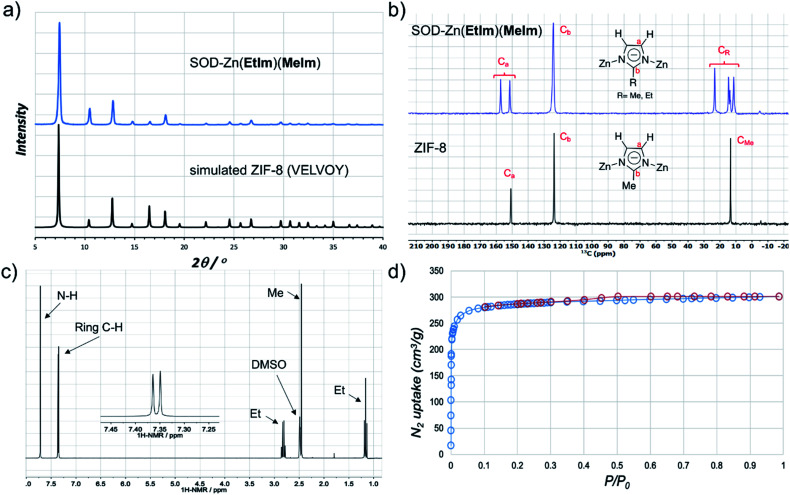
(a) PXRD pattern of SOD-Zn(**EtIm**)(**MeIm**) prepared by LA-RAM with CH_3_CN (1 hour, 95*g*, *η* ≈ 0.3 μL mg^−1^) compared to a simulated pattern for isostructural ZIF-8 (CSD code VELVOY); (b) solid-state ^13^C-NMR spectra for SOD-Zn(**EtIm**)(**MeIm**) (blue) and ZIF-8 (black) samples obtained by LA-RAM, after washing and evacuation (for solid-state ^15^N NMR spectra see ESI†); (c) ^1^H NMR spectrum for SOD-Zn(**EtIm**)(**MeIm**) after dissolution in DCl and DMSO-*d*_6_, illustrating the 1 : 1 ratio of **MeIm**^−^ and **EtIm**^−^ ligands (for relative signal integrations, see ESI†); (d) nitrogen desorption and adsorption curves for a washed and evacuated sample of SOD-Zn(**EtIm**)(**MeIm**) prepared by LA-RAM on ≈1.2 gram (5 mmol) scale.

The LA-RAM methodology was readily applied for the synthesis of a mixed-ligand ZIF system, by RAM of ZnO in the presence of a 1 : 1 stoichiometric mixture of 2-methyl- (H**MeIm**) and 2-ethylimidazole (H**EtIm**, Fig. 1). After one-hour of RAM at 95*g*, PXRD analysis of the reaction mixture revealed complete disappearance of ZnO, and the formation of a product isostructural to ZIF-8 (Fig. 3a) in quantitative conversion. As Zn(**EtIm**)_2_ framework is known to adopt *qtz*-, ANA- and RHO-topologies,^23^ this result indicates the formation of a mixed-ligand SOD-Zn(**EtIm**)(**MeIm**) framework. This was confirmed by TGA in air, as well as by solid-state NMR analysis of the washed and evacuated material, which clearly revealed the presence of both **MeIm**^−^ and **EtIm**^−^ ligands in the solid state (Fig. 3b). The composition of the material was additionally confirmed by dissolution of the washed material in D_2_O acidified with DCl. After further dilution with *d*_6_-DMSO, ^1^H NMR spectroscopy (Fig. 3c, see also ESI†) confirmed the 1 : 1 stoichiometric ratio of **MeIm**^−^ and **EtIm**^−^ ligands in the material. Importantly, performing the reaction of a 1 : 1 : 1 mixture of ZnO, H**MeIm** and H**EtIm** under otherwise identical conditions, but by ball milling, gave a SOD-topology product which, after washing and dissolution in acid, contained the two ligands in the respective stoichiometric ratio 60 : 40. This result, although preliminary, suggests that LA-RAM might provide for more efficient mixing than ball milling (see ESI†).

The synthesis of mixed-ligand SOD-Zn(**MeIm**)(**EtIm**) was also readily scaled five-fold (total expected amount of product *ca.* 1.2 grams), giving a phase-pure product based on PXRD analysis, with a TGA residue of 34.0% (compared to theoretically calculated 33.7%, see ESI†) and BET surface area of 1140 m^2^ g^−1^ (Fig. 3d and Table 1). Next, we screened the reactivity of a broader range of mixtures involving ZnO, H**EtIm** and H**MeIm**. The overall stoichiometric ratio of ZnO to total imidazole-based linker was kept at 1 : 2.1, respectively, and the stoichiometric ratio of H**MeIm** : H**EtIm** was varied between 0.15 : 1.95 and 0.35 : 1.75. Analysis of products by PXRD revealed the formation of separate phases (see ESI†) with SOD- and RHO-topology (CSD code MECWOH).

The described investigation of reactivity of ZnO and H**MeIm** at lower *g*-values revealed the formation of small amounts of a crystalline phase other than ZIF-8 (Fig. 2a). Comparison of X-ray reflections suggests the unexpected product is ZIF-L (see ESI†), a layered 2-D Zn(**MeIm**)_2_ framework containing additional neutral H**MeIm** ligands (CSD code IWOZOL).^13,14,24^ The observation of ZIF-L was particularly intriguing as this phase has not yet been observed in any reports on mechanochemical or solvent-free synthesis of ZIF-8, and has been significantly less explored compared to ZIF-8. Consequently, we explored the possibility of using RAM for targeted synthesis of ZIF-L. The observation of this phase under conditions of low acceleration guided us towards exploring milder RAM conditions, and using reactive metal precursors commonly used in solution synthesis: acetates or nitrates. While zinc acetate dihydrate Zn(OAc)_2_·2H_2_O was found to lead to very low conversions even in presence of a liquid (water), zinc nitrate hexahydrate Zn(NO_3_)_2_·6H_2_O was much more reactive (see ESI†). Analysis of a reaction mixture resulting from LA-RAM (acceleration of 30*g*, 30 minutes in presence of water) of Zn(NO_3_)_2_·6H_2_O and H**MeIm** in a stoichiometric ratio of 1 : 3.5, corresponding to the composition of ZIF-L, confirmed the formation of ZIF-L in a mixture with ZIF-8 (see ESI†). In order to optimize the reaction towards complete conversion to ZIF-L, the ratio of Zn precursor to H**MeIm** was increased. Complete conversion into ZIF-L was achieved at a Zn : H**MeIm** stoichiometric ratio of 1 : 8 (Fig. 2c), as evidenced by PXRD analysis, and TGA of the material after washing with water to remove excess H**MeIm** (see ESI†). The product was obtained, after washing and drying, in 86% isolated yield. Identical reaction conditions were also readily applicable for the synthesis of the Co(ii) version of ZIF-L,^25^ by using Co(NO_3_)_2_·6H_2_O as a metal precursor (see ESI†), providing the target material in 90% isolated yield after washing and drying. As ZIF-L is a very recently reported form of zinc 2-methylimidazolate^26^ we also considered it as a suitable target for RAM reaction scale-up, which proved to be straightforward, enabling one-pot syntheses of at least 25 grams of ZIF-L (Fig. 2d). To the best of our knowledge, this represents the first report of a bulk, multi-gram scale, as well as mechanochemical, synthesis of ZIF-L. Importantly, we were not able to obtain ZIF-L under identical conditions using ball milling, which yielded an as yet unidentified product (see ESI†). As ZIF-L is known to thermally transform to ZIF-8,^27^ its bulk synthesis further demonstrates the absence of significant thermal effects in LA-RAM synthesis.

**Fig. 4:**
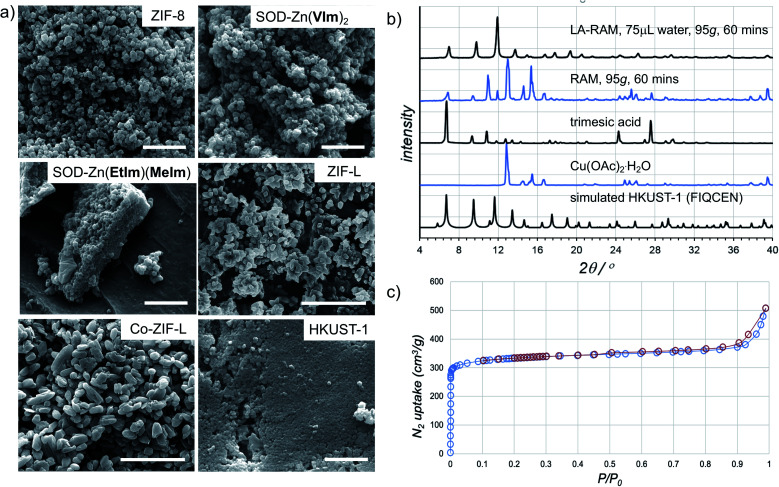
(a) Example SEM images for samples of herein prepared MOFs (in each case, the bar corresponds to 1 μm). (b) Comparison of the PXRD patterns for HKUST-1 prepared by LA-RAM with water (1 hour, 95*g*) to the neat reaction mixture, and the patterns of reactants and a simulated one for the MOF structure (CSD code FIQCEN). PXRD patterns for other LA-RAM experiments are given in the ESI.† (c) Nitrogen desorption and adsorption curves for HKUST-1 prepared by LA-RAM with water, after washing and evacuation.

The morphology of LA-RAM products was also studied by SEM, revealing hexagonal nanocrystalline particles of 50–200 nm size for Zn(**MeIm**)_2_ and Zn(**VIm**)_2_, and leaf-like particles with sizes ranging from 30–900 nm for ZIF-L (Fig. 4a).

In order to evaluate the applicability of RAM to other types of MOFs, we also explored the synthesis of the popular Cu(ii) trimesate framework material HKUST-1 (Fig. 1d), based on copper(ii) paddlewheel nodes.^28,29^ The mechanochemical synthesis of HKUST-1 by ball milling was previously reported by several groups, most often using copper(ii) acetate monohydrate Cu(OAc)_2_·H_2_O and trimesic acid as starting materials.^30,31^ Resonant acoustic mixing of a neat mixture of these reactants, in the stoichiometric ratio of 3 : 2 led to no reaction according to PXRD analysis. However, LA-RAM in the presence of diverse liquid additives produced HKUST-1, as evidenced by product PXRD patterns which in all cases exhibited Bragg reflections consistent with those of the MOF (CSD code FIQCEN).^28^ In most cases, the PXRD pattern also exhibited additional X-ray reflections (see ESI†), indicative of impurities or side products. We believe that these, so far not identified phases, could result from the liquid additive coordinating to the metal center and/or templating a different type of framework structure.^32^ Indeed, we have recently shown that choice of milling additive can have a strong effect on mechanochemical LAG synthesis of zirconium MOFs due to coordination properties of the liquid.^33^ Nevertheless, using water as the liquid additive led to the formation of HKUST-1 as the only product, as evidenced by PXRD and TGA (Fig. 4b, also see ESI†). The HKUST-1 made by RAM (95*g*, 1 hour, *η* = 0.15 μL mg^−1^) was obtained, after washing and evacuation, in 88% isolated yield. The material exhibited a surface area of 1310 m^2^ g^−1^ (Fig. 4c), consistent with previously reported values (see ESI†).^29,30^

## Conclusions

The use of resonant acoustic mixing as a simple, rapid and readily scalable methodology for synthesizing metal–organic frameworks of different levels of complexity, including two- and three-dimensional networks, has been demonstrated. This methodology is based on high-frequency acoustic agitation, does not use bulk solvent, and in contrast to other mechanochemical routes for MOF synthesis it does not require any milling or grinding media, enabling simple and straightforward scaling-up of batch synthesis from hundreds of milligrams to at least tens of grams, while still permitting the use of a metal oxide as a starting material. Importantly, the herein explored model systems demonstrate the ability to use acoustic mixing not only for making conventional, well-explored MOF materials such as ZIF-8 or HKUST-1, but also more complex mixed-ligand microporous solids, as well as the metastable^27^ layered material ZIF-L, that has previously never been reported by mechanosynthesis. We believe that the ability to form the ZIF-L product might be associated to LA-RAM being a milder mechanochemical methodology, in which mechanical activation takes place by direct contact of reactant particles rather than through impact and abrasion by external milling media that are used in other types of mechanochemistry. Importantly, in each of these cases LA-RAM methodology appears to be superior to ball milling, which provided poorer control of product composition in the synthesis of a mixed-ligand MOF, and also did not yield ZIF-L. Further uses of LA-RAM as a general and easily scalable route to other types of functional materials, and the ability to use liquid additives and catalysts to enhance reactions in an acoustically-agitated solid-state environment, are currently being explored.

## Conflicts of interest

There are no conflicts to declare.

## Supplementary Material

SC-011-D0SC00333F-s001

SC-011-D0SC00333F-s002
