# Supplementary material for: Simple, scalable mechanosynthesis of metal–organic frameworks using liquid-assisted resonant acoustic mixing (LA-RAM)
Source: Chem Sci. 2020 Feb 27;11(29):7578–84. doi: 10.1039/d0sc00333f (PMC8159441; doi:10.1039/d0sc00333f)
Supplement: SC-011-D0SC00333F-s001 [file SC-011-D0SC00333F-s001.pdf]

Electronic Supplementary Material (ESI) for Chemical Science.
This journal is © The Royal Society of Chemistry 2020

Simple, scalable mechanosynthesis of metal–organic frameworks using liquid-assisted resonant acoustic mixing (LA-RAM)

Hatem M. Titi,[a] Jean-Louis Do,[a,b] Ashlee J. Howarth,[b] Karthik Nagapudi,[c] and Tomislav Friščić[a]*

Table of Contents

Materials and methods	2
Synthesis	3
Selected characterization data	5
Powder X-ray diffraction (PXRD)	5
Thermal analysis data	15
Fourier-transform infrared attenuated total reflectance spectroscopy (FTIR-ATR)	20
Nuclear magnetic resonance (NMR) spectroscopy	22
Solid-state nuclear magnetic resonance (ssNMR) spectroscopy	23
N_2 sorption isotherms for surface area measurements	24
References	25

1. Materials and methods

All reactions were performed under an atmosphere of argon unless otherwise specified. Acetonitrile, methanol (MeOH), ethanol (EtOH), acetonitrile (CH_3CN), N,N-dimethylformamide (DMF), acetone, *iso*-propanol, and *n*-propanol were obtained from a PureSolv™ PS-400 solvent purification system. Zinc oxide, ammonium nitrate, copper(II) acetate monohydrate ($Cu(OAc)_2{\cdot}H_2O$), zinc nitrate hexahydrate ($Zn(NO_3)_2{\cdot}6H_2O$), cobalt(II) nitrate hexahydrate ($Co(NO_3)_2{\cdot}6H_2O$), trimesic acid, 2-ethylimidazole (H**EtIm**) and 2-methylimidazole (H**MeIm**) were obtained from Sigma-Aldrich. The ligand 2-vinylimidazole (H**VIm**) was synthesized according to a reported procedure.[1]

1.1. Nuclear magnetic resonance (NMR)

Solution ^{1}H-NMR spectra (500 MHz) were recorded on Bruker Ascend™ spectrometer. Chemical shifts are reported relative to DMSO-d6 (δ 2.50 ppm) for ^{1}H-NMR spectra. NMR samples of the commercial ZIF-8, H**EtIm**, H**MeIm**, and Zn(**EtIm**)(**MeIm**) were prepared by the addition of 200 μL of DCl and DMSO-d_6. The samples were sonicated until complete dissolution. The ^{1}H NMR data are presented as follows: chemical shift, multiplicity and integration.

1.2. Solid-state nuclear magnetic resonance (ssNMR)

Solid-state cross-polarization magic angle spinning (CP-MAS) ^{13}C- and ^{15}N-NMR spectra were acquired on a Varian VNMRS 400 MHz NMR spectrometer operating at 100.53 MHz for ^{13}C, 40.51MHz for ^{15}N and at 399.77 MHz for ^{1}H using a wide-bore 4-mm T3 double-resonance probe spinning at 13 kHz for ^{13}C and 8 kHz for ^{15}N. Cross-polarization using RAMP CP (ramped-amplitude cross-polarization) for 2 ms ^{13}C and 3 ms for ^{15}N. SPINAL-64 ^{1}H decoupling was performed during acquisition using a 90-kHz rf field. Recycle delays are 3s for ^{13}C and 10s for ^{15}N, as number of scans for ZIF-8 is 64 scans for ^{13}C and 10000 scans for nitrogen, and for the mixed ligand was 1600 scans for ^{13}C and 5200 scans for nitrogen.

1.3. Fourier-transform infrared attenuated total reflectance spectroscopy (FTIR-ATR)

Infrared spectra were obtained using a Bruker Vertex 70 FTIR spectrometer equipped with the Platinum ATR accessory. Absorption bands are reported in wavenumbers (cm^{-1}).

1.4. Thermal analysis

Thermogravimetric analysis and differential scanning calorimetry (DSC) data were measured on a TGA/DSC 1 (Mettler-Toledo, Columbus, Ohio, USA) instrument. TGA of SOD-Zn(**EtIm**)(**MeIm**) was measured on a TGA250 (TA instruments Ltd., Delaware, USA). Samples (5 to 10 mg) were placed in alumina crucibles. All measurements were done under a 25 mL min^{-1} stream of air, and the samples were heated from RT up to 800 °C using a constant heating ramp of 10 °C min^{-1}.

1.5. Powder X-ray diffraction (PXRD)

Powder X-ray diffraction (PXRD) data were collected on a Bruker D2 Phaser diffractometer equipped with a LYNXEYE linear position sensitive detector (Bruker AXS, Madison, WI, USA), using Ni-filtered Cu*K*α radiation.

1.6. Scanning electron microscopy (SEM)

SEM images were recorded on a QUANTA FEG 450 electron microscope. Evacuated MOF samples were loaded on carbon tape by *via* dispersion in hexanes and were sputter-coated with 4 nm of platinum before measurement.

1.7. Surface area measurements (BET)

Nitrogen adsorption and desorption isotherm measurements were performed on a Micromeritics TriStar II Plus instrument at a temperature of 77 K.

2. Synthesis

2.1. Synthesis of SOD-Zn(MeIm)$_2$ (ZIF-8) and SOD-Zn(VIm)$_2$

A mixture of ZnO (1 mmol, 81 mg), H**MeIm** (2.1 mmol, 173 mg) or H**VIm** (2.1 mmol, 198 mg) and NH_4NO_3 (4 mg , 5 mol% with respect to zinc), and 75 µL of MeOH were added to an 8 mL plastic vial, and the mixture oscillated for 60 minutes at 95 *g* to yield a microcrystalline product, as confirmed by PXRD. The product was washed/centrifuged at 4,500 rpm with 15 mL EtOH three times. The product was evacuated overnight at 85 °C.

2.2. Scaling-up SOD-Zn(MeIm)$_2$ (ZIF-8)

A mixture of ZnO (5 mmol, 405 mg), H**MeIm** (10.5 mmol, 861 mg), NH_4NO_3 (20 mg), and 500 µL CH_3CN was added to an 8 mL plastic vial. The mixture was oscillated for 60 minutes at 95 *g* to yield a microcrystalline product, as confirmed by PXRD. The product was washed/centrifuged at 4,500 rpm with 15 mL EtOH three times. The product was evacuated overnight at 85 °C.

2.3. Synthesis of ZIF-L and Co-ZIF-L

A mixture of $Zn(NO_3)_2{\cdot}6H_2O$ (1 mmol, 298 mg) or $Co(NO_3)_2{\cdot}6H_2O$ (1 mmol, 291 mg), H**MeIm** (8 mmol, 656 mg) and 25 µL water were added to an 8 mL plastic vial. The mixture was oscillated for 30 minutes at 30 *g* to yield a microcrystalline product, as confirmed by PXRD. The product was washed with 15 mL of water, followed by centrifugation at 4,500 rpm for 15 mins, three times.

2.4. Scale-up of ZIF-L synthesis to 25 grams

A mixture of $Zn(NO_3)_2{\cdot}6H_2O$ (0.1 mol, 30 gram), H**MeIm** (0.8 mol, 66 gram) and 7.5 mL water was added to a 100 mL plastic vial. The mixture was placed in the RAM for 30 minutes at oscillation of 30 *g* to yield a microcrystalline product, as confirmed by PXRD.

The product was washed with 30 mL of water, followed by centrifugation at 4,500 rpm for 15 mins, three times.

2.5. Synthesis of SOD-Zn(EtIm)(MeIm)

A mixture of ZnO (1 mmol, 81 mg), H**EtIm** (1.05 mmol, 101 mg), H**MeIm** (1.05 mmol, 86 mg), NH_4NO_3 (4 mg, 5 mol%), and 75 µL CH_3CN was added to an 8 mL plastic vial. The mixture was oscillated for 60 minutes at 95 *g* to yield a microcrystalline product, as evidenced by PXRD. The product was washed with 15 mL MeOH, followed by centrifugation at 4,500 rpm for 15 mins, three times. The dry product was evacuated overnight at 85 °C.

2.6. Scaling-up SOD-Zn(EtIm)(MeIm)

A mixture of ZnO (5 mmol, 405 mg), H**EtIm** (5.3 mmol, 510 mg), H**MeIm** (5.3 mmol, 435 mg) and NH_4NO_3 (20 mg) and 375 µL CH_3CN were added to an 8 mL plastic vial. The mixture was oscillated for 60 minutes at 95 *g* to yield a microcrystalline product, as evidenced by PXRD analysis. The product was washed with 30 mL MeOH, followed by centrifugation at 4,500 rpm for 15 mins, three times. The dry product was evacuated overnight at 85 °C.

2.7. Synthesis of SOD-Zn(EtIm)(MeIm) by ball milling

A mixture of ZnO (1 mmol, 81 mg), H**EtIm**H (1.05 mmol, 101 mg), H**MeIm** (1.05 mmol, 86 mg), NH_4NO_3 (4mg, 5 mol%), and 75 µL CH_3CN were added to a 15 mL stainless-steel milling jar with two stainless-steel milling balls (1.34 gram, 7mm diameter each). The mixture was milled for 30 minutes at a frequency of 30 Hz using a Retsch MM400 shaker mill, to yield a microcrystalline product, evidenced by PXRD. The product was washed with 15 mL MeOH, followed by centrifugation at 4,500 rpm for 15 mins, three times. The dry product was evacuated overnight at 85 °C.

2.8. Synthesis of HKUST-1

A mixture of $Cu(OAc)_2 \cdot H_2O$ (1.5 mmol, 300mg) and trimesic acid (1 mmol, 210 mg) was placed in an 8 mL plastic vial, along with either 75 µL, 125 µL and 150 µL of a liquid additive (Table S1). The mixtures were oscillated at 95 *g* for 1 hour and analyzed by PXRD. Formation of clean HKUST-1 was observed with water as the liquid additive, and the product was further washed with MeOH and evacuated at 100 °C for two days.

Table S1: Synthesis of HKUST-1 using RAM

REACTION	MILLING MEDIA	LIQUID ADDITIVE
1	-	EtOH (125 μL)
2	ten 3mm ZrO_2 beads	EtOH (125 μL)
3	-	MeOH (125 μL)
4	-	CH_3CN (125 μL)
5	-	DMF (150 μL)
6	-	*n*-propanol (150 μL)
7	-	*iso*-propanol (150 μL)
8	-	EtOH (150 μL)
9	-	water (75 μL)

3. Selected characterization data

3.1. Powder X-ray diffraction (PXRD)

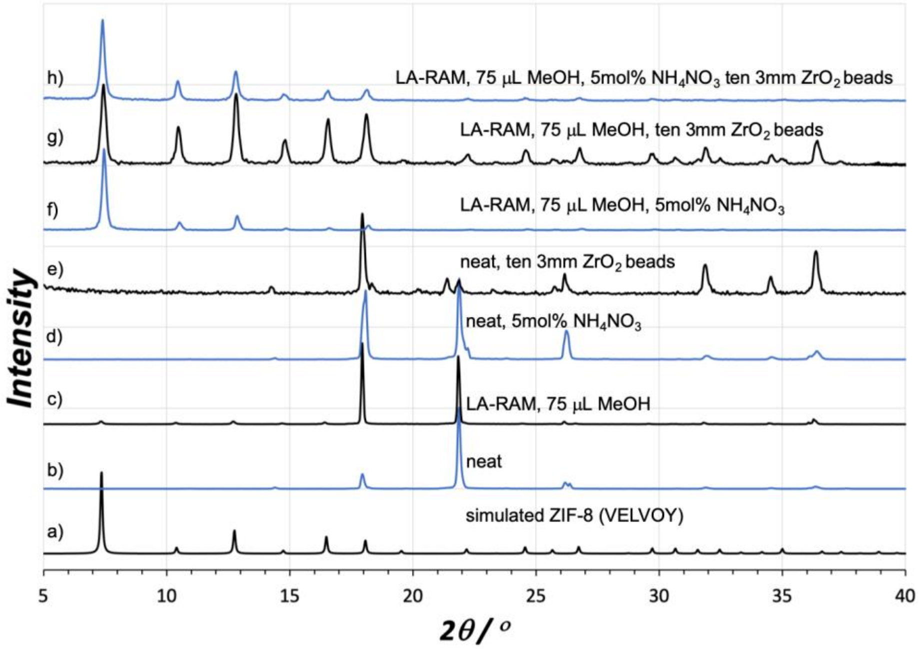

Fig. S1. Comparison of PXRD patterns for crude products of SOD-Zn(**MeIm**)$_2$ (ZIF-8) synthesis using RAM: a) simulated pattern for the structure of ZIF-8; b) neat oscillation; c) LA-RAM with 75 μL of MeOH ($\eta \approx 0.3$ μL/mg); d) neat oscillation with 5 mol% NH_4NO_3; e) oscillation with ten ZrO_2 beads of 3 mm diameter; f) LA-RAM with 75 μL of MeOH and 5 mol% NH_4NO_3 ($\eta \approx 0.3$ μL/mg); g) LA-RAM with 75μL MeOH and ten 3mm ZrO_2 beads ($\eta \approx 0.3$ μL/mg); h) LA-RAM with 75μL MeOH, 5 mol% NH_4NO_3 and ten 3 mm ZrO_2 beads ($\eta \approx 0.3$ μL/mg). All mixtures contained ZnO and H**MeIm** in the respective 1:2.1 stoichiometric ration, and were oscillated at 95 *g* for 60 minutes.

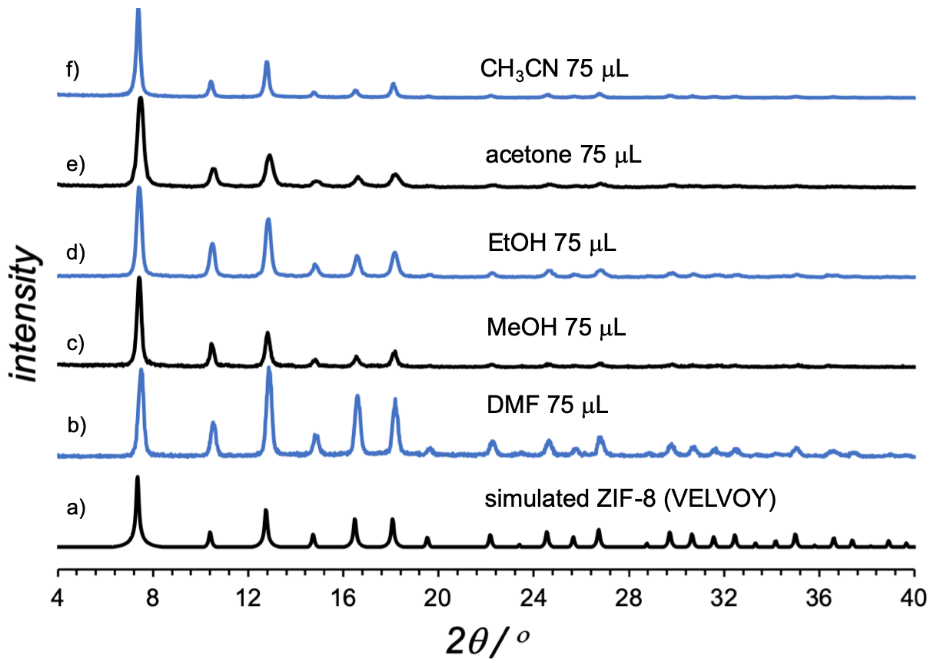

Fig. S2. Comparison of PXRD patterns for SOD-Zn(**MeIm**)$_2$ (ZIF-8) obtained by LA-RAM using different liquid additives (75 µL, $\eta \approx 0.3$ µL/mg), in presence of 5 mol% NH_4NO_3: a) simulated for ZIF-8; b) with DMF; c) with MeOH; d) with EtOH; e) with acetone and f) with CH_3CN. All mixtures contained ZnO and H**MeIm** in the respective 1:2.1 stoichiometric and were oscillated at 95 *g* for 60 minutes.

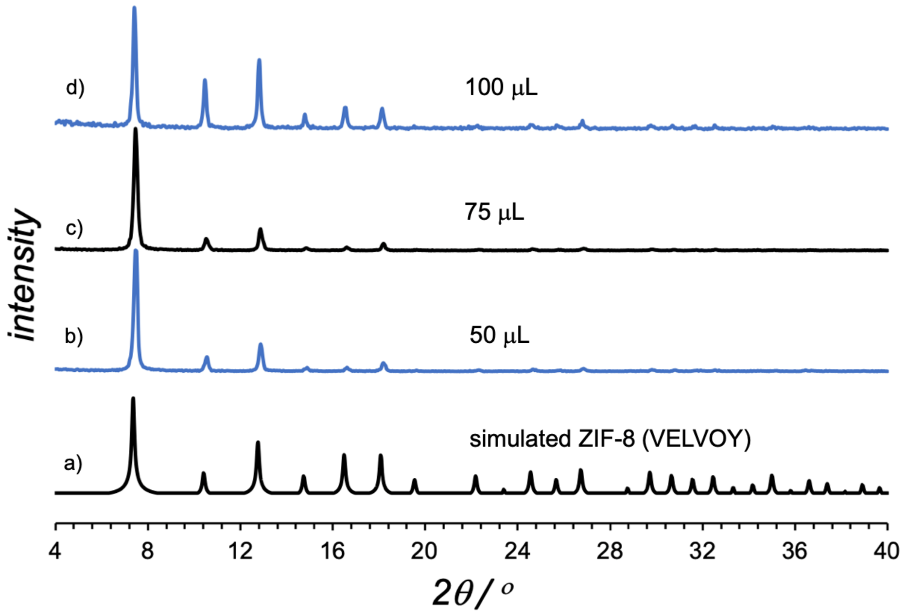

Fig. S3. Comparison of PXRD patterns for SOD-Zn(**MeIm**)$_2$ (ZIF-8) obtained by LA-RAM with different amounts of MeOH and in presence of 5 mol% NH_4NO_3: a) simulated pattern for ZIF-8; b) LA-RAM with 50 µL MeOH ($\eta \approx 0.2$ µL/mg); c) LA-RAM with 75 µL MeOH ($\eta \approx 0.3$ µL/mg) and d) LA-RAM with 100 µL MeOH ($\eta \approx 0.4$ µL/mg). All mixtures contained ZnO and H**MeIm** in the respective stoichiometric ratio 1:2.1 and were oscillated at 95 *g* for 60 minutes.

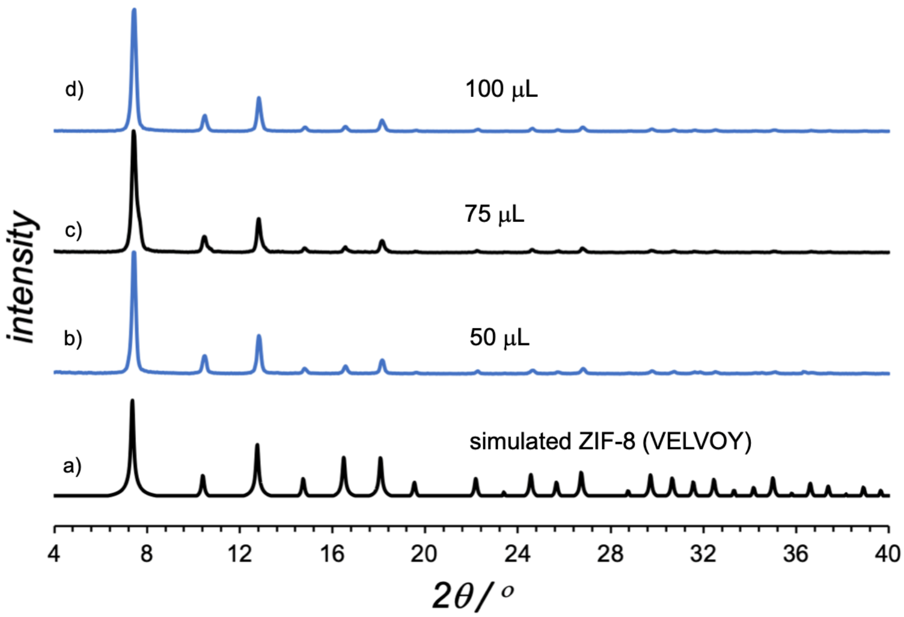

Fig. S4. Comparison of PXRD patterns for SOD-Zn(**MeIm**)$_2$ (ZIF-8) obtained by LA-RAM using different amounts of MeOH and in presence of 5 mol% NH_4NO_3: a) simulated pattern for ZIF-8; b) LA-RAM with 50 µL MeOH ($\eta \approx 0.2$ µL/mg); c) LA-RAM with 75 µL MeOH ($\eta \approx 0.3$ µL/mg) and d) LA-RAM with 100 µL MeOH ($\eta \approx 0.4$ µL/mg). All mixtures contained ZnO and H**MeIm** in the respective stoichiometric ratio 1:2.1 and were oscillated at 75 *g* for 60 minutes.

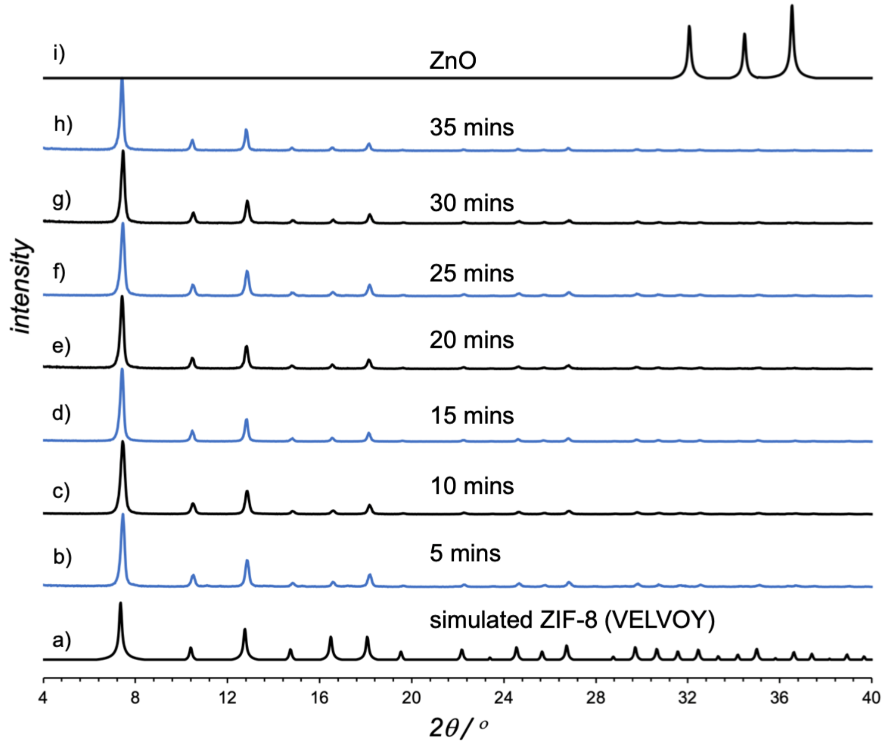

Fig. S5. Comparison of PXRD patterns for the synthesis of SOD-Zn(**MeIm**)$_2$ (ZIF-8) by LA-RAM at different times: a) simulated for ZIF-8; b) 5 minutes; c) 10 minutes; d) 15 minutes; e) 20 minutes; f) 25 minutes; g) 30 minutes; h) 35 minutes; i) ZnO reactant. All reactions were performed at 95 *g* and contained a stoichiometric 1:2.1 mixture of ZnO and H**MeIm**, 75 µL MeOH and 5 mol% NH_4NO_3 ($\eta \approx 0.3$ µL/mg).

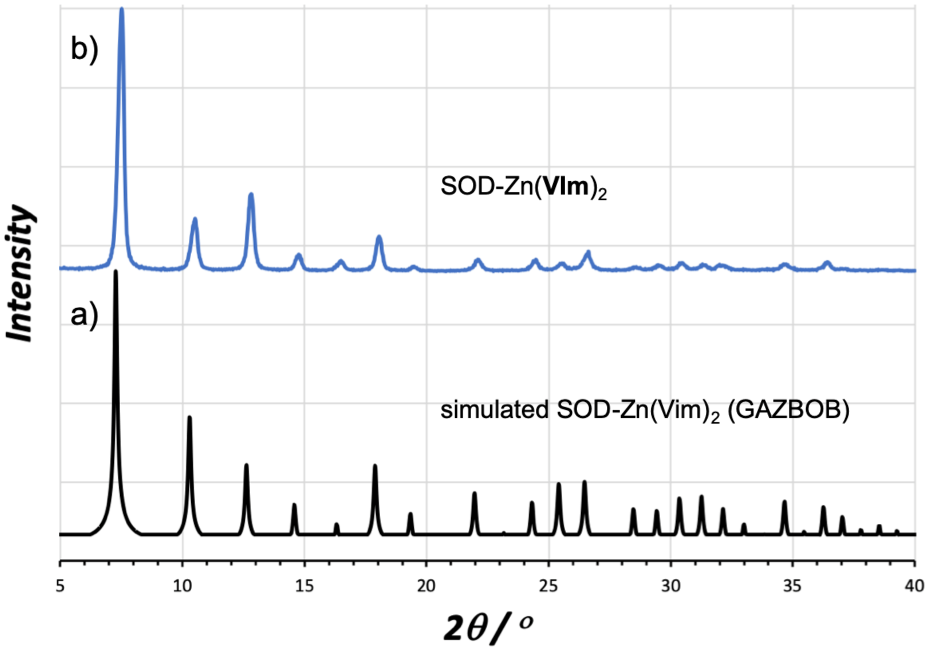

Fig. S6. Comparison of the PXRD patterns: a) SOD-Zn(**VIm**)$_2$ obtained by LA-RAM of a 1:2.1 stoichiometric mixture of ZnO and H**MeIm** with 75 μL MeOH and 5 mol% NH_4NO_3; b) simulated pattern for SOD-Zn(**VIm**)$_2$. The reaction was oscillated for 60 minutes at 95 *g*.

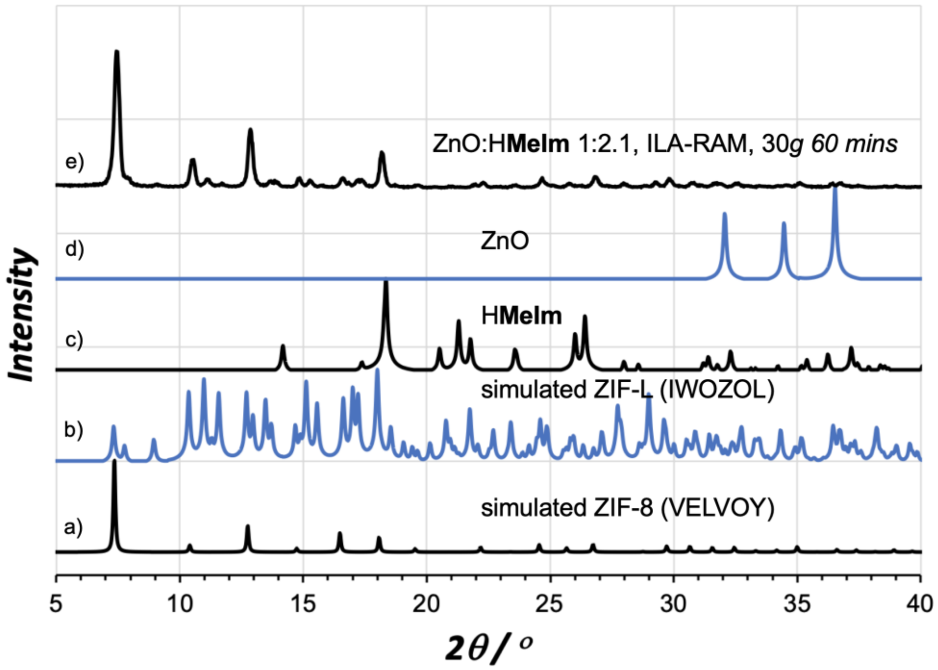

Fig. S7. Comparison of PXRD patterns for attempted synthesis of SOD-Zn(**MeIm**)$_2$ (ZIF-8) by LA-RAM at 30 *g*, with 75 μL MeOH and 5 mol% NH_4NO_3 ($\eta \approx 0.3$ μL/mg): a) simulated for ZIF-8; b) simulated for ZIF-L; c) H**MeIm** reactant; d) ZnO reactant and e) reaction mixture after 60 minutes oscillation.

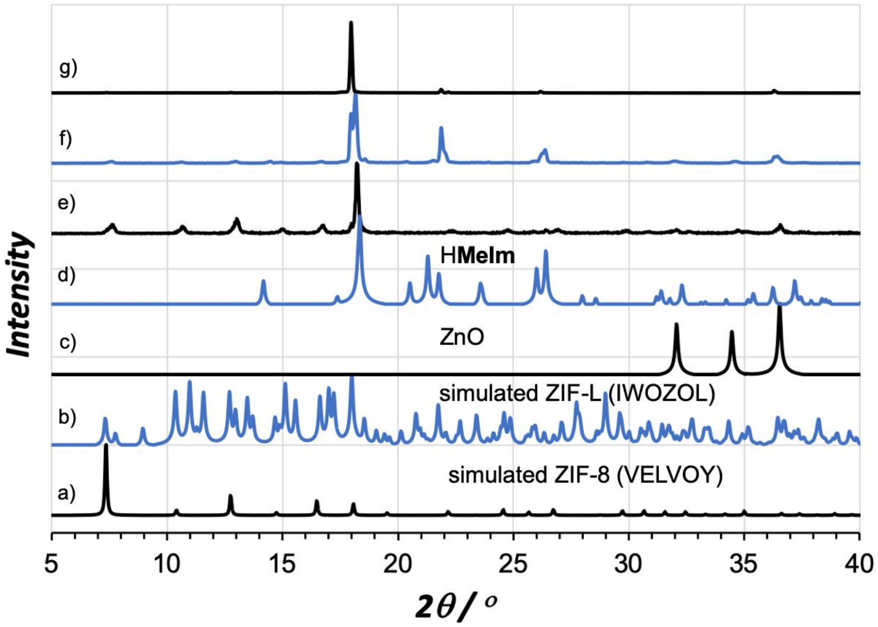

Fig. S8. Selected PXRD patterns for attempted synthesis of ZIF-L: a) simulated for ZIF-8; b) simulated for ZIF-L; c) ZnO reactant; d) H**MeIm** reactant; e) mixture of ZnO and H**MeIm** in the respective stoichiometric ratio 1:2.1, along with 5 mol% of NH_4NO_3 and 75 μL MeOH, after 5 minutes at 30 *g* ($\eta \approx 0.3$ μL/mg); f) mixture of ZnO and H**MeIm** in the respective stoichiometric ratio 1:8, with 75 μL water, after 30 minutes at 30 *g*; g) mixture of $Zn(OAc)_2{\cdot}2H_2O$ and H**MeIm** in respective stoichiometric ratio 1:8, with 75 μL water, after 30 minutes at 30 *g*.

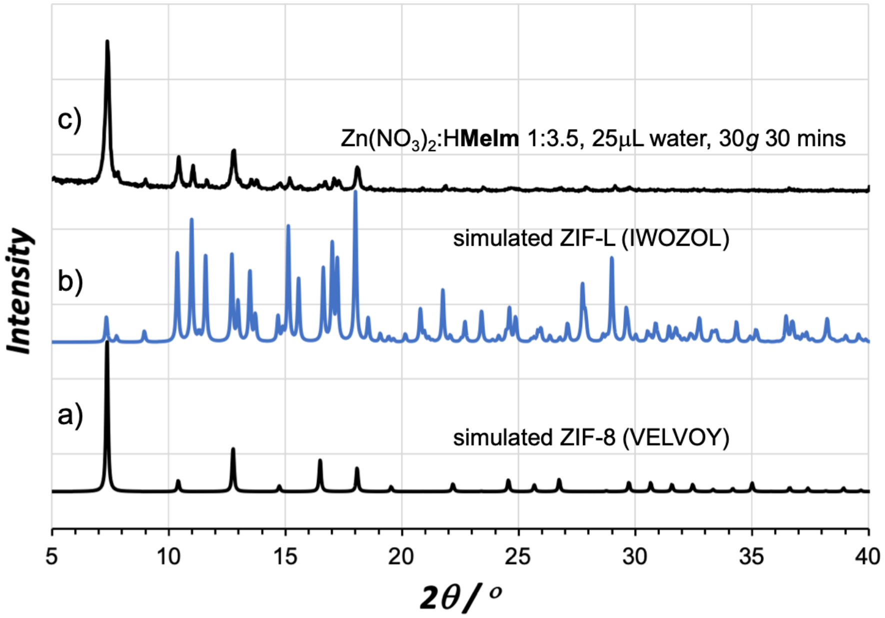

Fig. S9. Example PXRD patterns for attempted synthesis of ZIF-L from $Zn(NO_3)_2{\cdot}6H_2O$ and H**MeIm** in respective stoichiometric ratio 1:3.5 by LA-RAM with 25 μL water: a) simulated for ZIF-8; b) simulated for ZIF-L and c) reaction mixture after 30 minutes at 30 *g*.

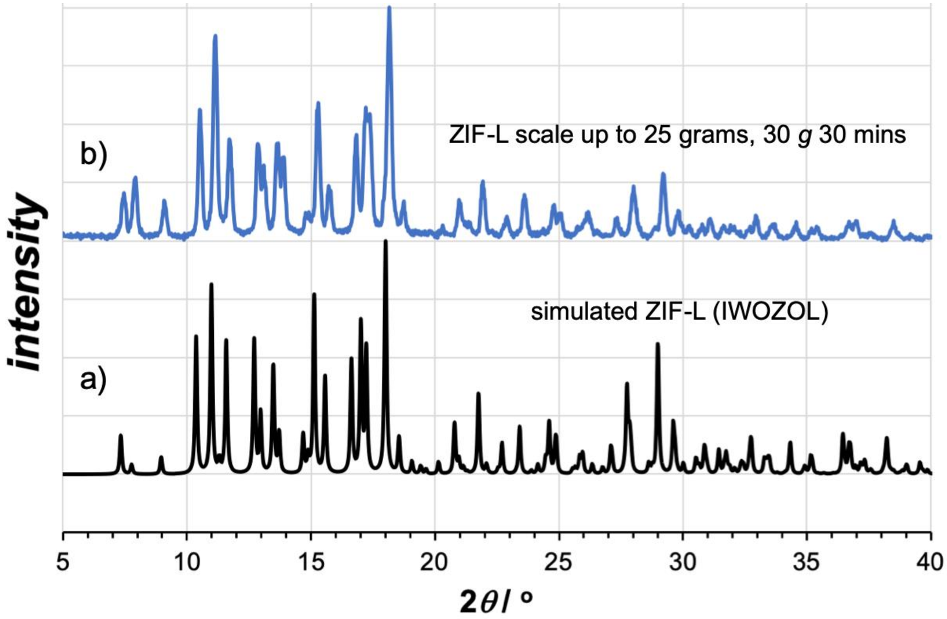

Fig. S10. PXRD patterns for ZIF-L prepared by LA-RAM: a) simulated for ZIF-L (CSD code IWOZOL) and b) product obtained at 25 gram scale.

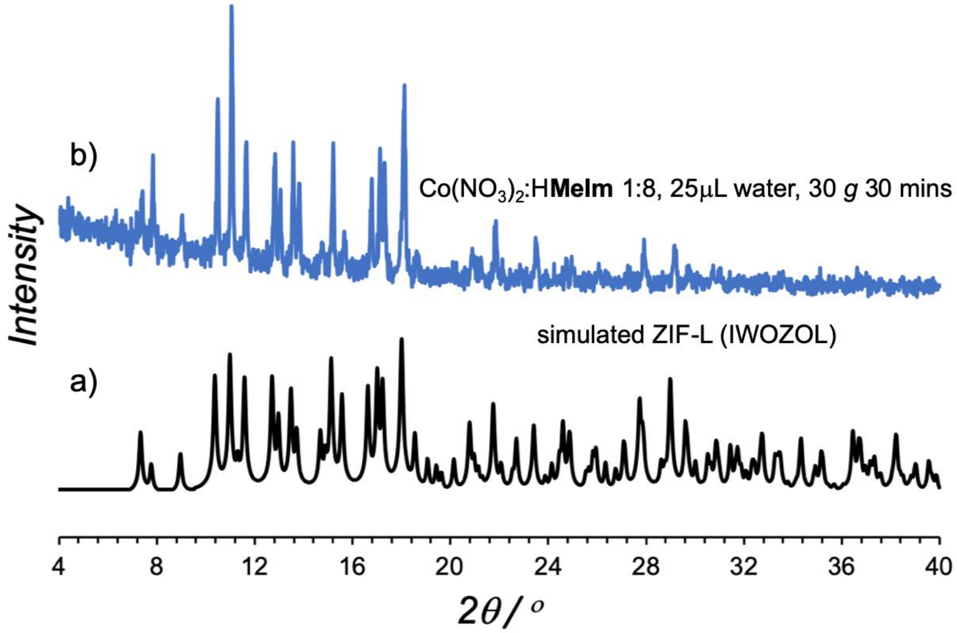

Fig. S11. PXRD patterns for Co-ZIF-L synthesized by LA-RAM: a) simulated pattern of ZIF-L (CSD code IWOZOL); b) product obtained after 30 minutes at 30 *g* using $Co(NO_3)_2 \cdot 6H_2O$ and H**MeIm**, in respective stoichiometric ratio 1:8, in the presence of 25 mL of water. The measured PXRD pattern is of poor quality due to strong fluorescence of the cobalt-containing product in CuK_α radiation.

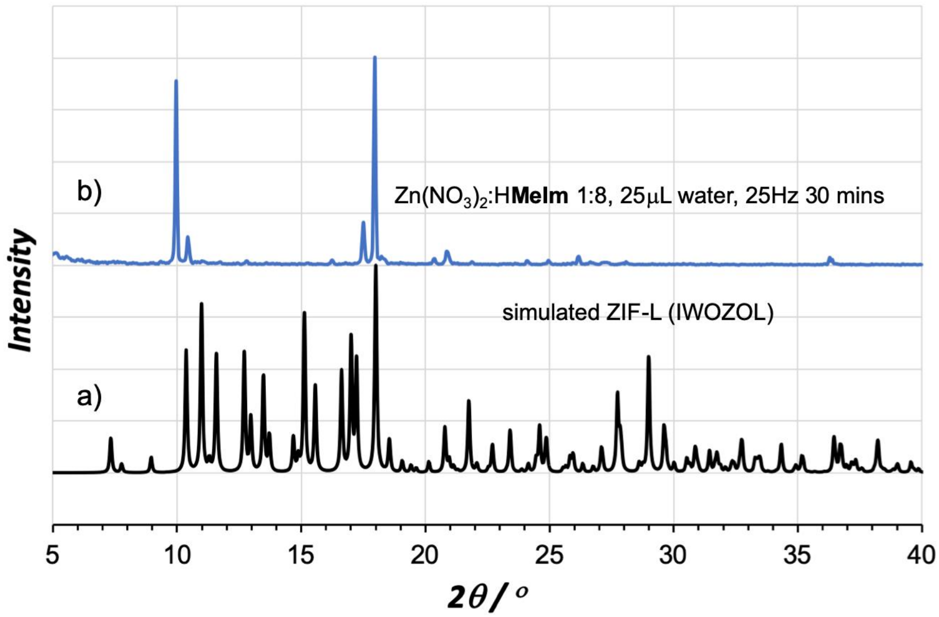

Fig. S12. PXRD patterns for attempted synthasis of ZIF-L by ball milling: a) simulated for ZIF-L (CSD code IWOZOL) and b) product of ball milling a mixture of $Zn(NO_3)_2 \cdot 6H_2O$ and H**MeIm** in the respective stoichiometric ratio 1:8, in the presence of 25 μL water, for 30 minutes at 25 Hz. Milling was done using two stainless steel balls of 7 mm diameter.

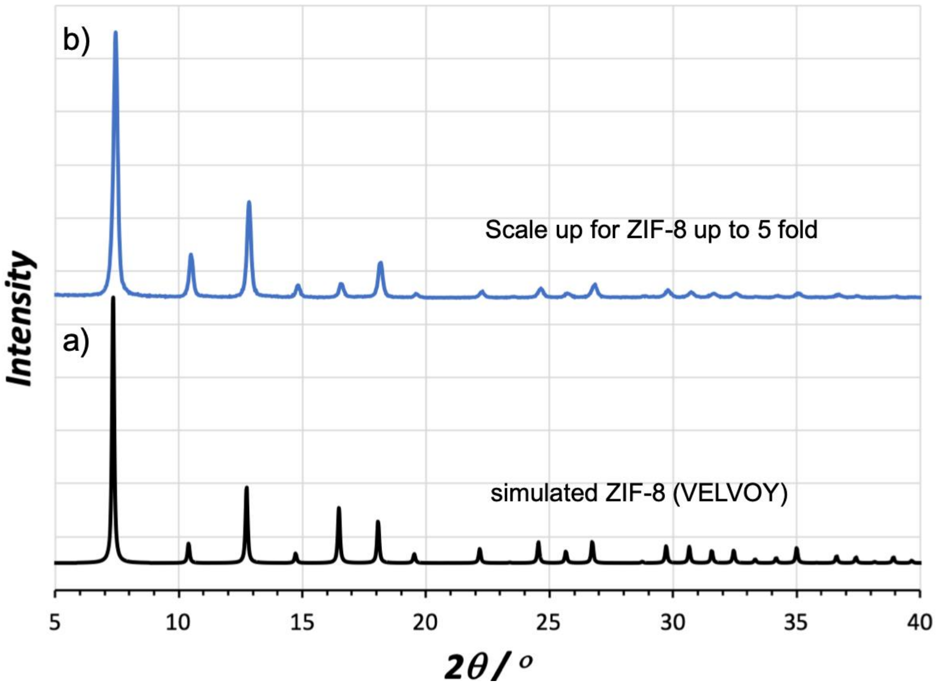

Fig. S13. PXRD patterns for scaling-up of the ZIF-8 synthesis to 1 gram: a) simulated for ZIF-8 and b) washed and evacuated sample, obtained on 1.2 gram scale by LA-RAM in the presence of NH_4NO_3 and CH_3CN as the liquid additive ($\eta \approx 0.3$ μL/mg), after 60 minutes at 95 *g*.

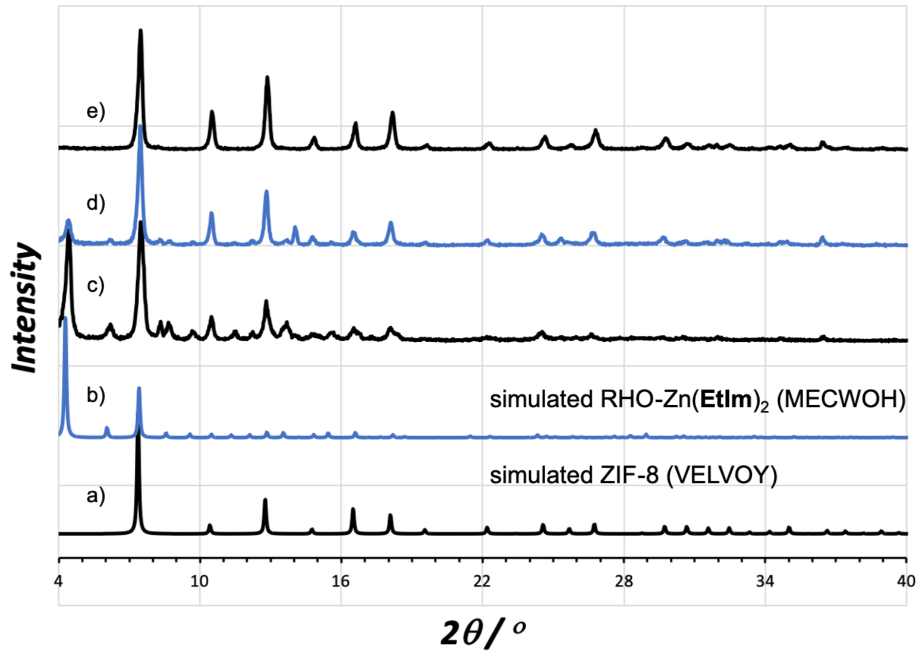

Fig. S14. Comparison of PXRD patterns for LA-RAM reactions containing ZnO and different ratios of H**MeIm** and H**EtIm**. Simulated for reference ZIFs with a) SOD- (CSD code VELVOY) and b) RHO-topology (CSD code MECWOH). Reaction mixtures after 60 minutes at 95 *g* for the H**MeIm**:H**EtIm** stoichiometric ratios: c) 0.15:1.95; d) 0.35:1.75 and e) 1.05:1.05. All reactions were performed in presence of 5 mol% NH_4NO_3 and 75 μL CH_3CN as the liquid additive ($\eta \approx 0.3$ μL/mg), with the relative stoichiometric ratio of ZnO to total imidazoles always 1:2.1.

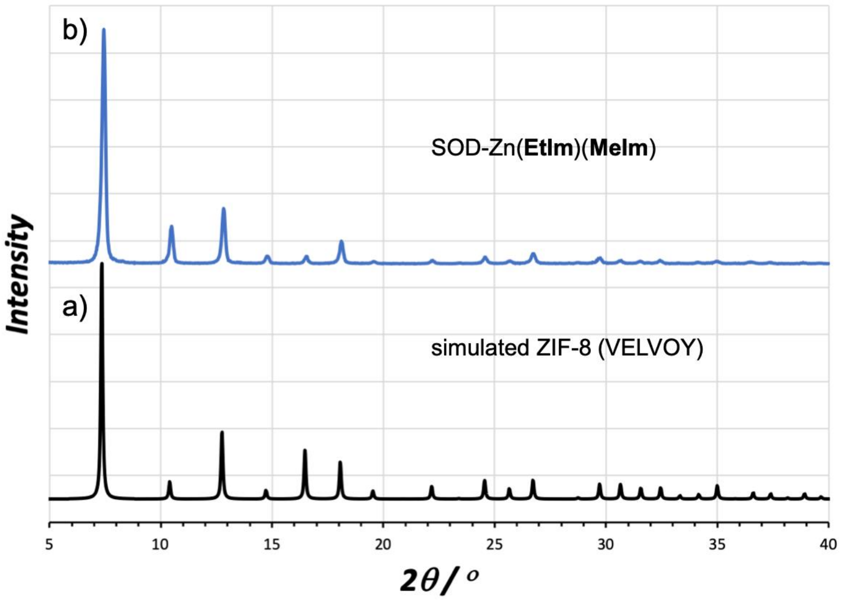

Fig. S15. PXRD patterns for SOD-Zn(**EtIm**)(**MeIm**) obtained by RAM: a) simulated for ZIF-8 and b) a washed and evacuated sample synthesized by LA-RAM of a mixture of ZnO, H**MeIm** and H**EtIm** in the relative stoichiometric ratio 1:1.05:1.05, in presence of 75 μL CH_3CN and 5 mol% NH_4NO_3 ($\eta \approx 0.3$ μL/mg), at 95 *g* for 60 minutes.

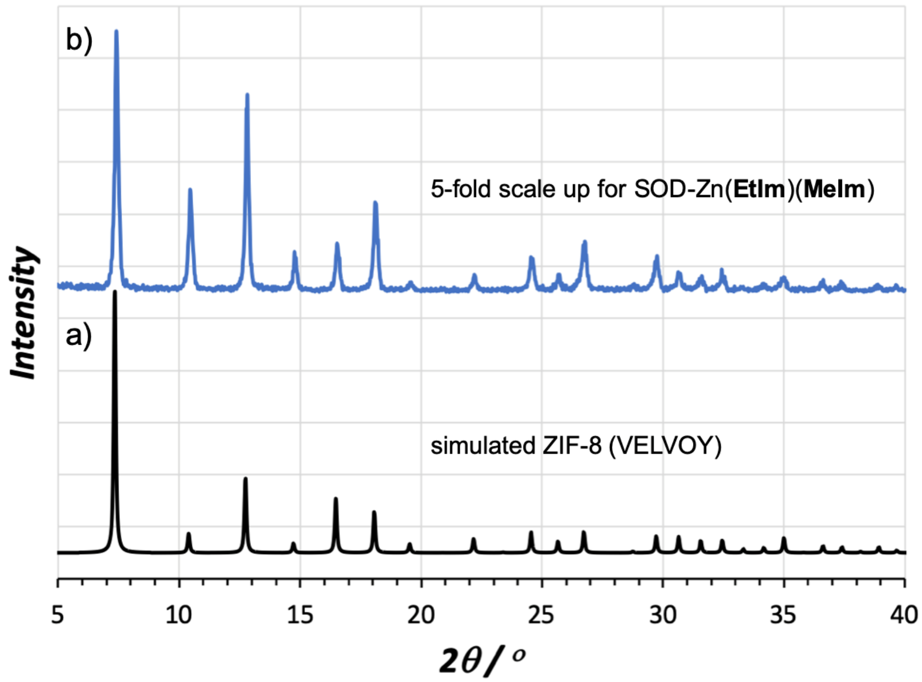

Fig. S16. PXRD patterns scaling-up of the synthesis of SOD-Zn(**EtIm**)(**MeIm**) to 1 gram: a) simulated for ZIF-8 and b) a washed and evacuated sample of SOD-Zn(**MeIm**)(**EtIm**) obtained on 1.2 gram scale by LA-RAM in the presence of NH_4NO_3 and CH_3CN as the liquid additive ($\eta \approx 0.3$ μL/mg). The reaction was performed by at 95 *g* for 60 minutes.

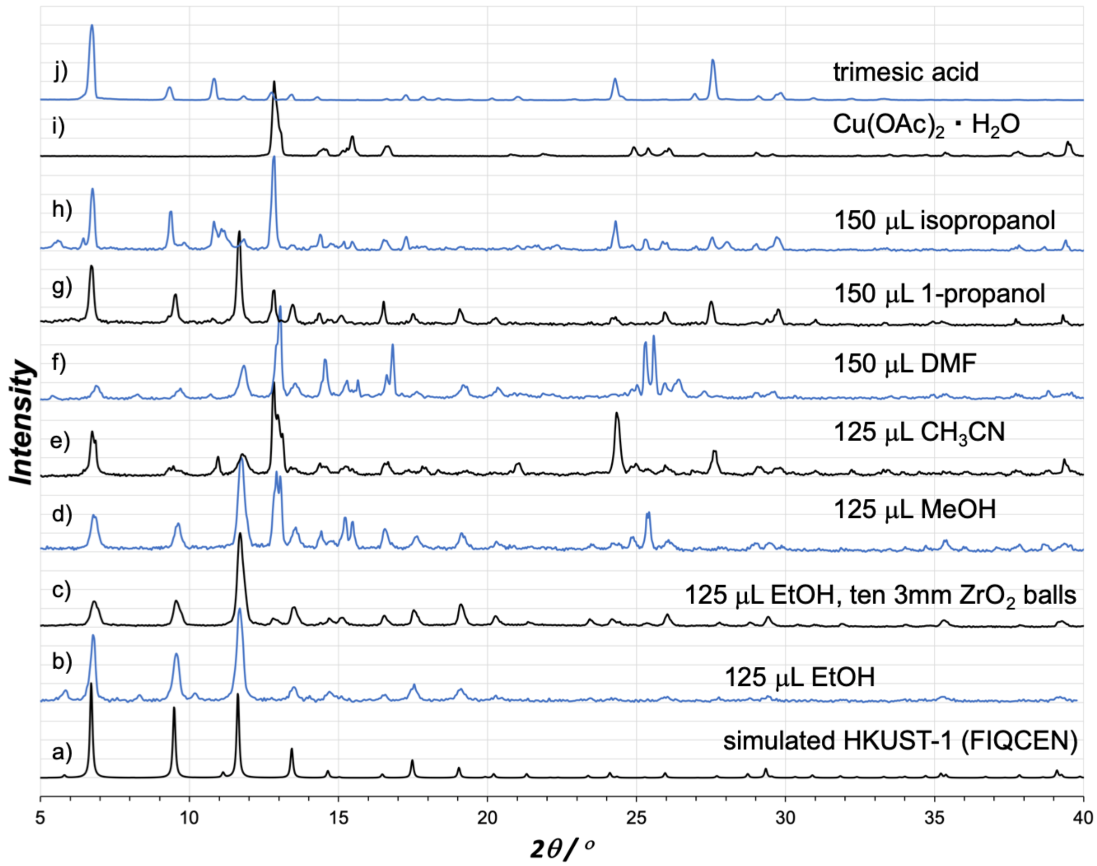

Fig. S17. Comparison of PXRD patterns for products of attempted HKUST-1 synthesis by LA-RAM of $Cu(OAc)_2{\cdot}H_2O$ and trimesic acid in stoichiometric ratio 3:2 by LA-RAM in the presence of different liquid additives: a) simulated for the MOF structure (CSD code FIQCEN); b) with 125 μL EtOH ($\eta \approx$ 0.25 μL/mg); c) with 125 μL EtOH and ten ZrO_2 beads of 3 mm diameter ($\eta \approx$ 0.25 μL/mg); d) with 125 μL MeOH ($\eta \approx$ 0.25 μL/mg); e) with 125 μL CH_3CN ($\eta \approx$ 0.25 μL/mg); f) with 150 μL DMF ($\eta \approx$ 0.29 μL/mg); g) with 150 μL *n*-propanol ($\eta \approx$ 0.29 μL/mg); h) with 150 μL *i*-propanol ($\eta \approx$ 0.29 μL/mg); i) $Cu(OAc)_2{\cdot}H_2O$ reactant and j) trimesic acid reactant. All reaction mixtures obtained after 60 minutes at 95 *g*. Formation of HKUST-1 was observed in all cases, but quantitative conversion was observed only with water as the additive (see manuscript, Figure 4).

3.2. Thermal analysis data

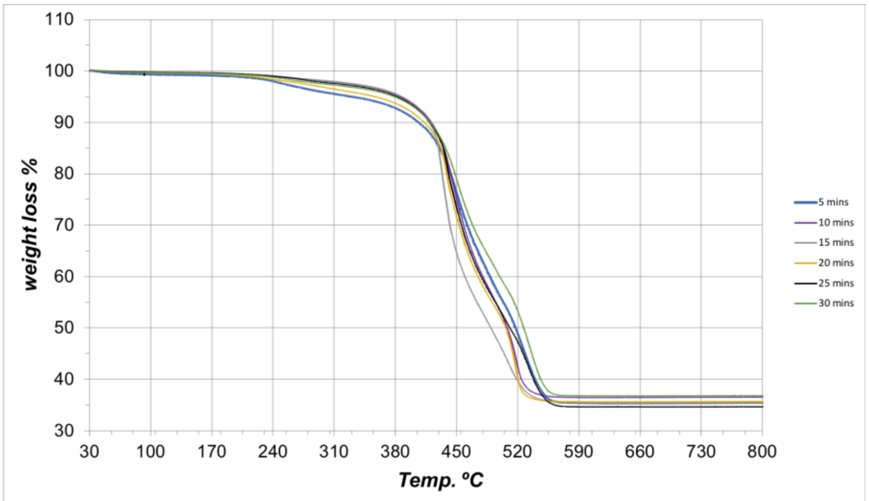

Fig. S18. Comparison of TGA thermograms for washed and evacuated samples of ZIF-8 obtained by LA-RAM (75 μL MeOH, in presence of 5 mol% NH_4NO_3) at 95 *g* for different times (5, 10, 15, 20, 25 and 30 mins).

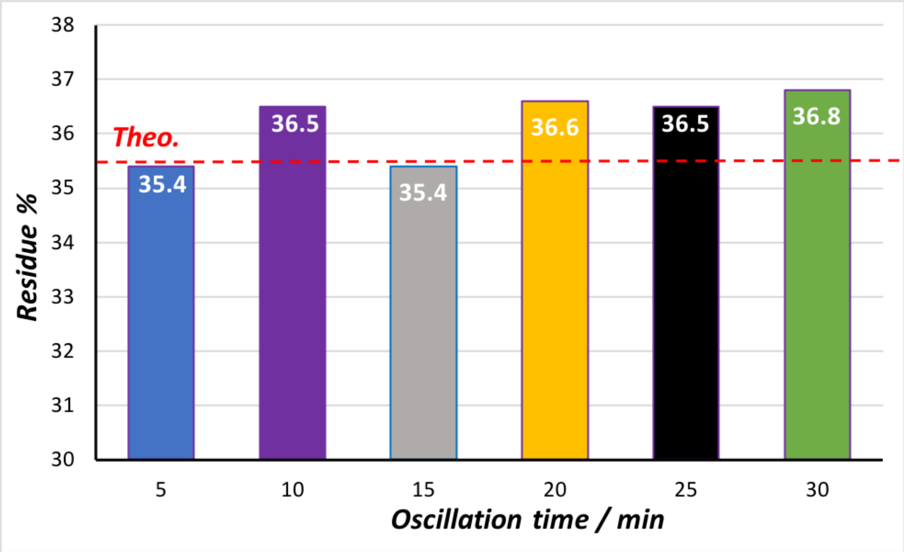

Fig. S19. Comparison of residues from TGA of washed and evacuated samples of ZIF-8 obtained by LA-RAM (75 μL MeOH, in presence of 5 mol% NH_4NO_3) at 95 *g* for different times (5, 10, 15, 20, 25 and 30 mins). Theoretically expected ZnO residue is indicated by the red dashed line.

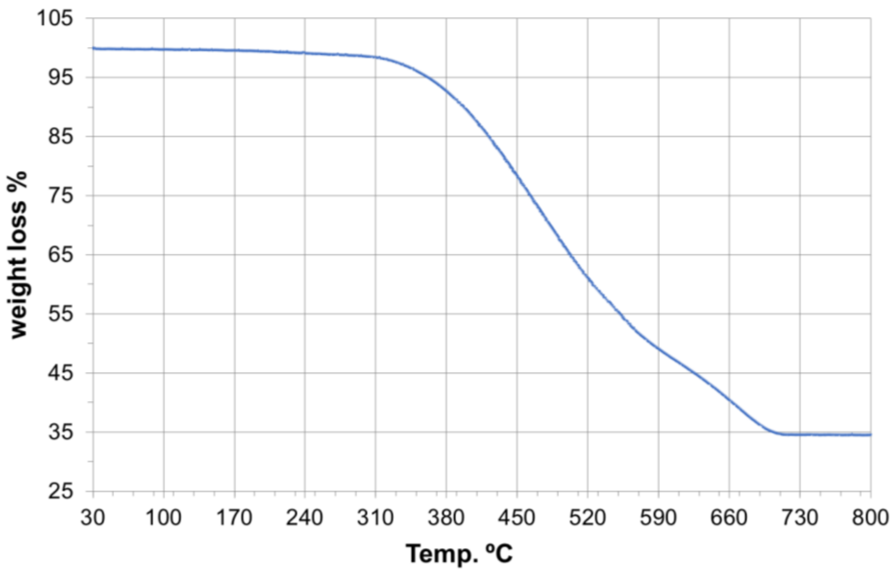

Fig. S20. TGA thermogram of a sample of washed and evacuated SOD-Zn(**VIm**)$_2$. The residue is 34.6% (expected 32.3 %)

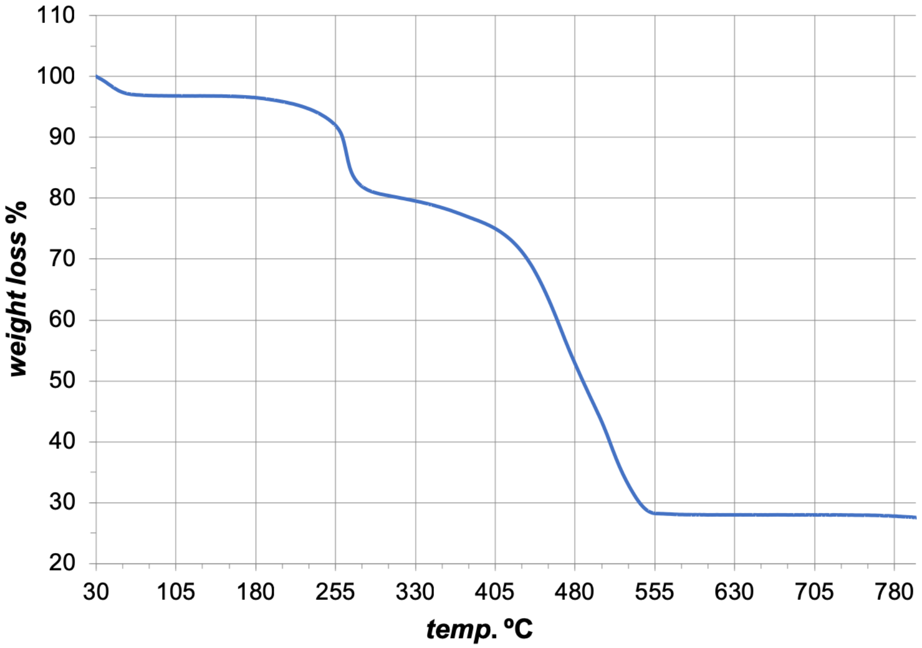

Fig. S21. TGA thermogram of a washed and evacuated sample of ZIF-L. The residue is 28.0% (expected 27.8%).

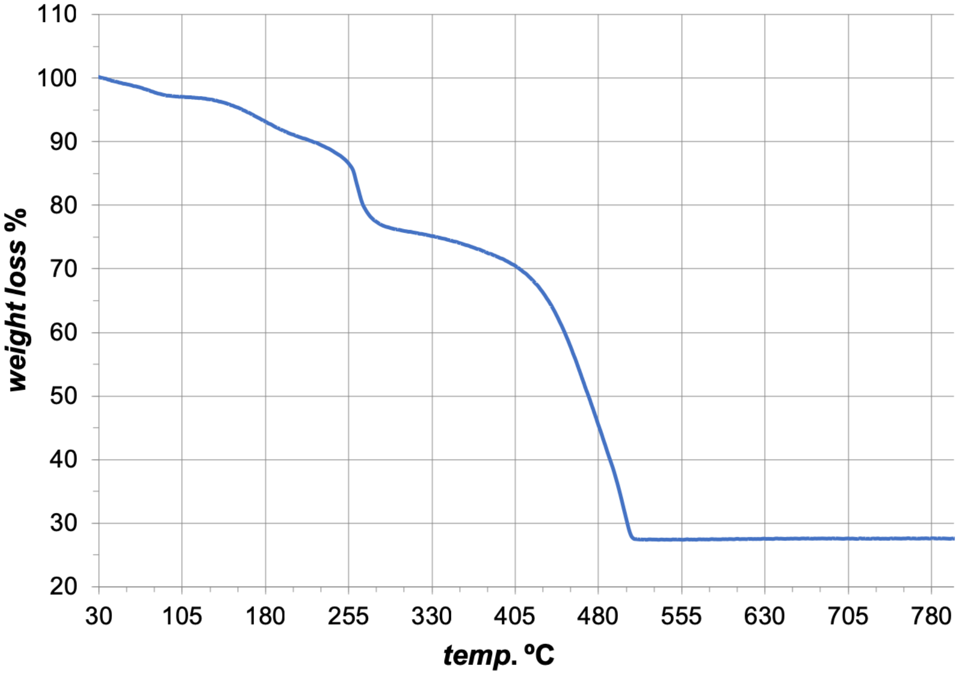

Fig. S22. TGA thermogram of ZIF-L obtained in 25 gram scale, the compound was washed with water and kept at 70 °C for dryness. The residue is 27.4% (expected 27.8 %).

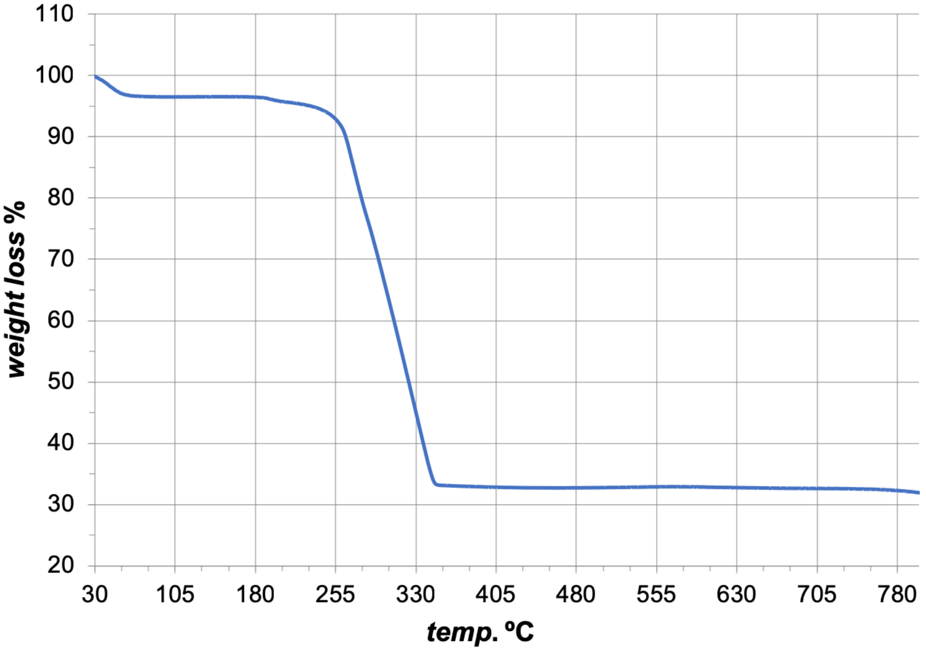

Fig. S23. TGA thermogram of a washed and evacuated sample of Co-ZIF-L. The residue is 32.7% (calculated 30.6% based on Co_3O_4).

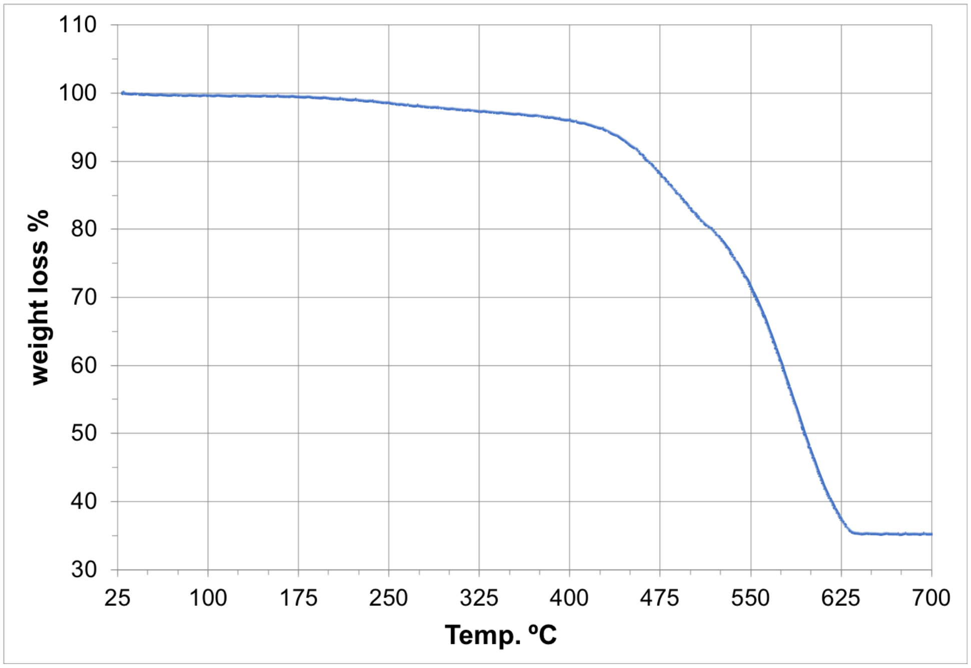

Fig. S24. TGA thermogram of a scale up (5 fold, ≈ 1.2 g) reaction of SOD-Zn(**MeIm**)$_2$ after being washed and evacuated, the residue found 35.3% (calc. 35.5%).

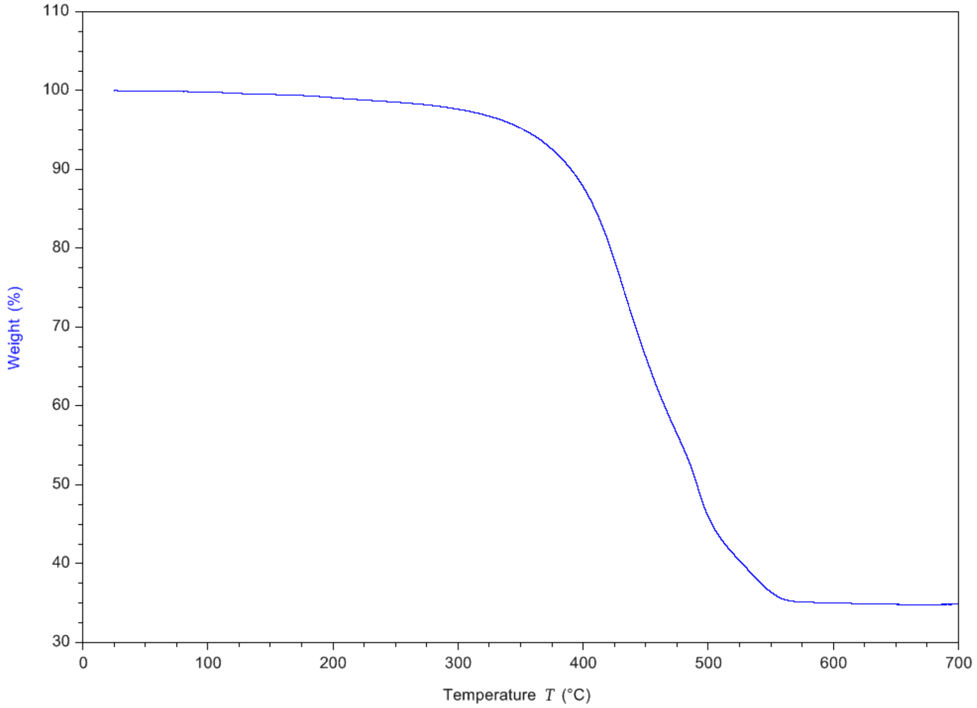

Fig. S25. TGA thermogram of washed and evacuated SOD-Zn(**EtIm**)(**MeIm**), Exp: 34.9% (Calc: 33.7%)

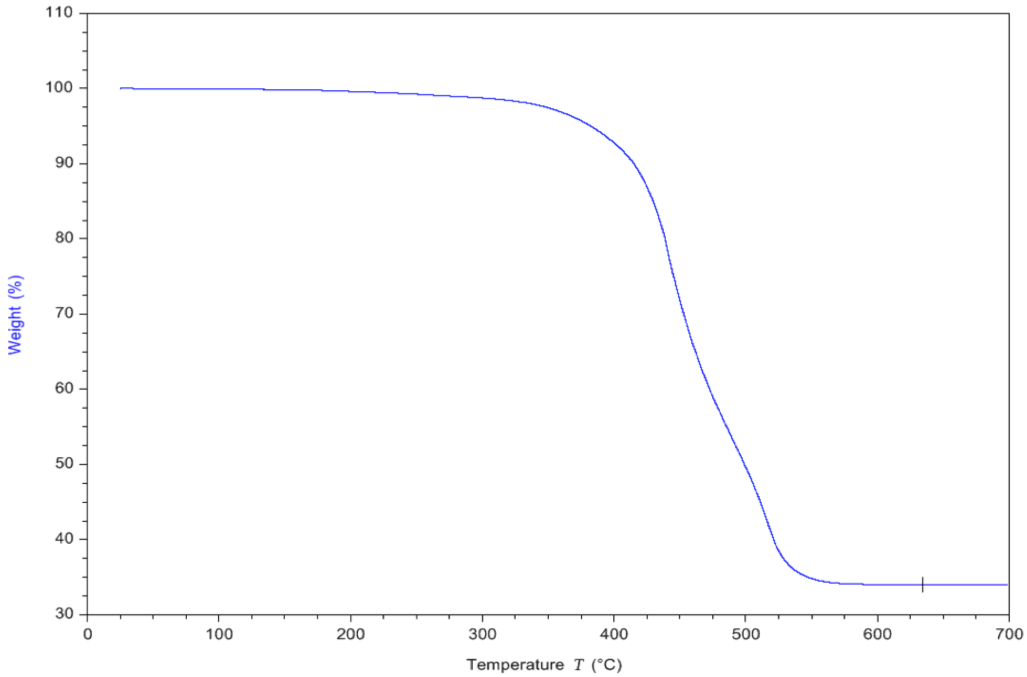

Fig. S26. TGA thermogram of washed and evacuated SOD-Zn(**EtIm**)(**MeIm**) for 5 fold reaction ca. 1 gram, the residue found 34.0% (Calc: 33.7%)

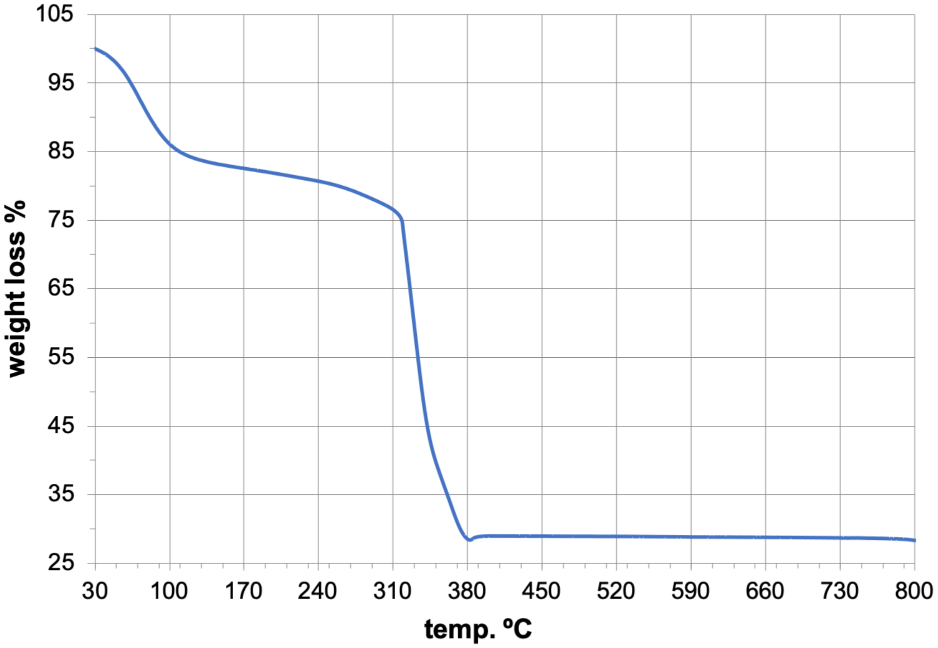

Fig. S27. TGA thermogram of HKUST-1 prepared by LA-RAM with water, after washing evacuation, residue found 34.1% (expected 32.4%).

3.3. Fourier-transform infrared attenuated total reflectance spectroscopy (FTIR-ATR)

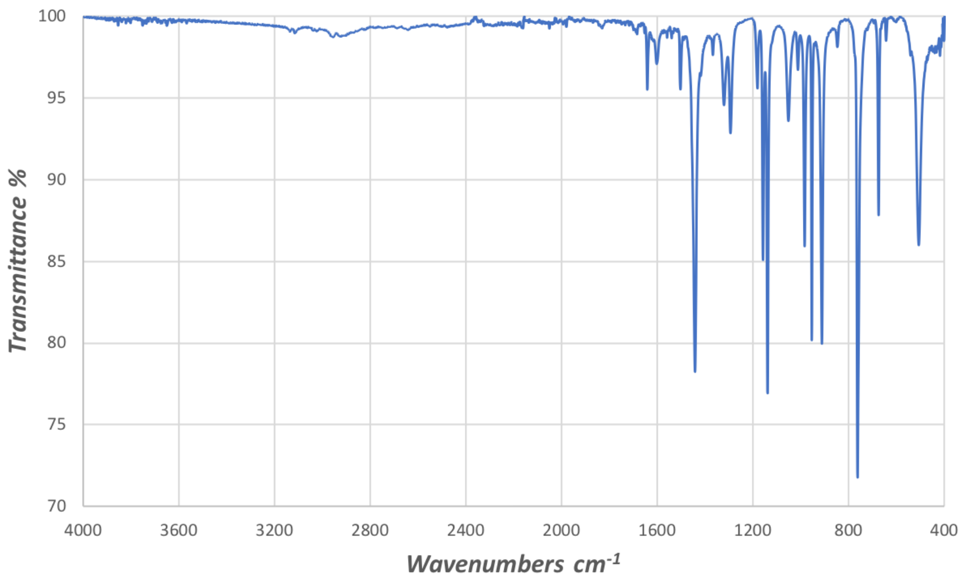

Fig. S28. FTIR spectrum of SOD-Zn(**VIm**)$_2$ after washing and evacuation.

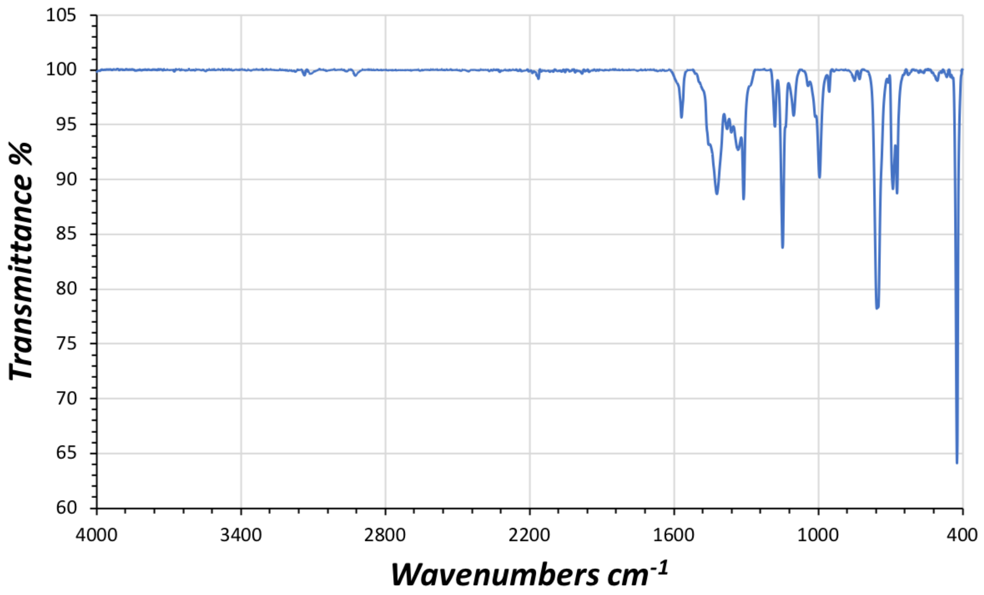

Fig. S29. FTIR-ATR spectrum of a sample of ZIF-L.

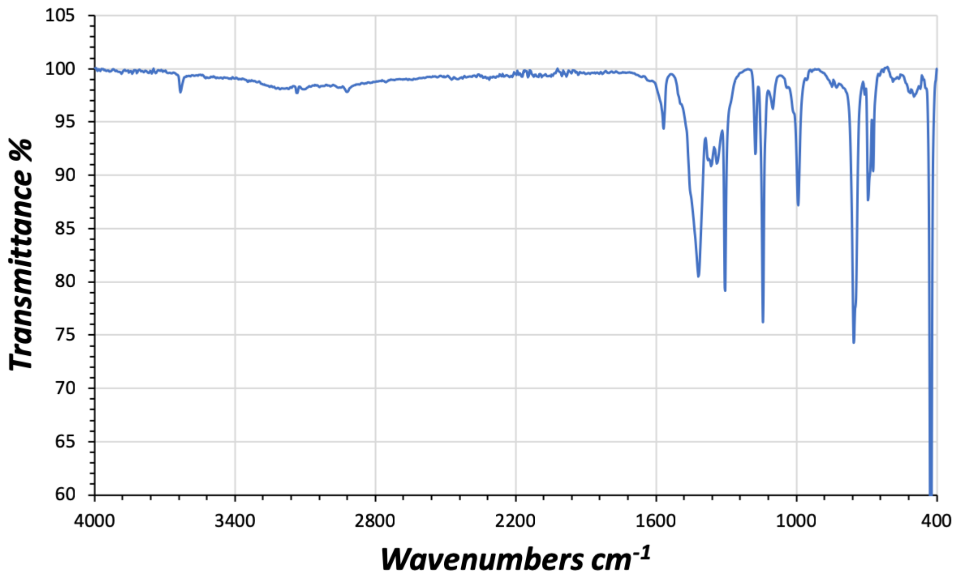

Fig. S30. FTIR-ATR spectrum for Co-ZIF-L.

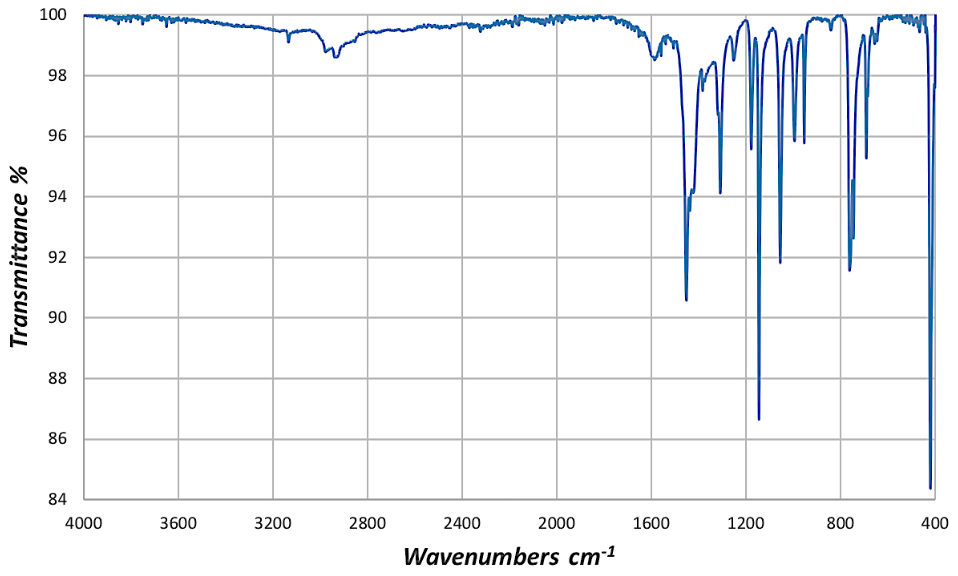

Fig. S31. FTIR-ATR spectrum of the mixed-ligand MOF SOD-Zn(**EtIm**)(**MeIm**), after washing and evacuation.

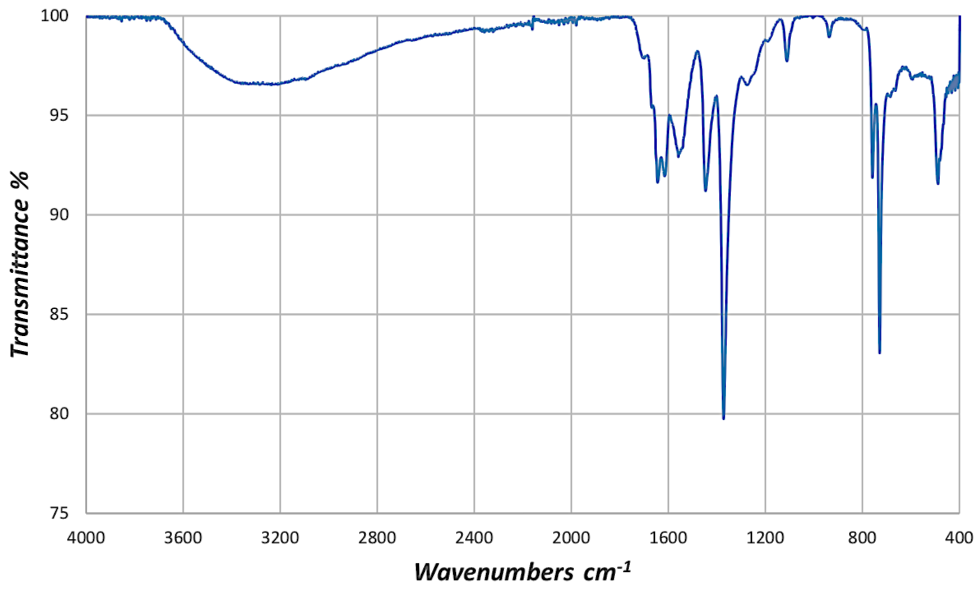

Fig. S32. FTIR-ATR spectrum of HKUST-1 prepared by LA-RAM using water as the liquid additive.

3.4. Nuclear magnetic resonance (NMR) spectroscopy

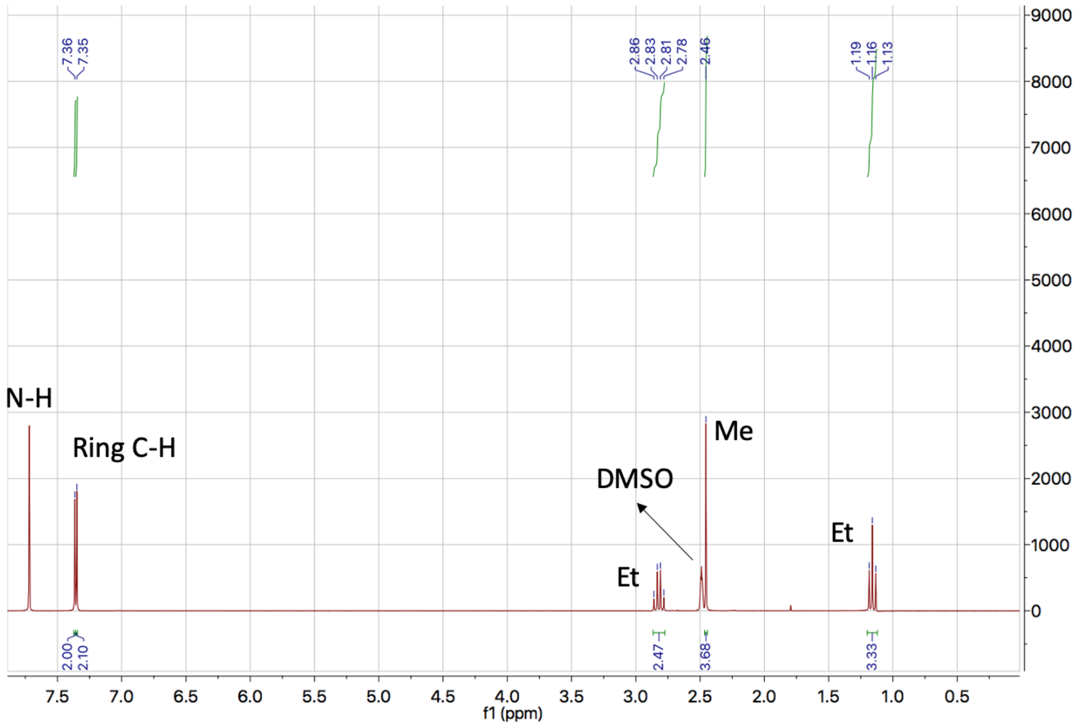

Fig. S33. ^{1}H-NMR spectrum of a washed and evacuated sample of SOD-Zn(**EtIm**)(**MeIm**) prepared by LA-RAM, after dissolution in DCl and DMSO-d_6. The integration of signals indicates the stoichiometric ratio **MeIm**:**EtIm** of ca. 50:50.

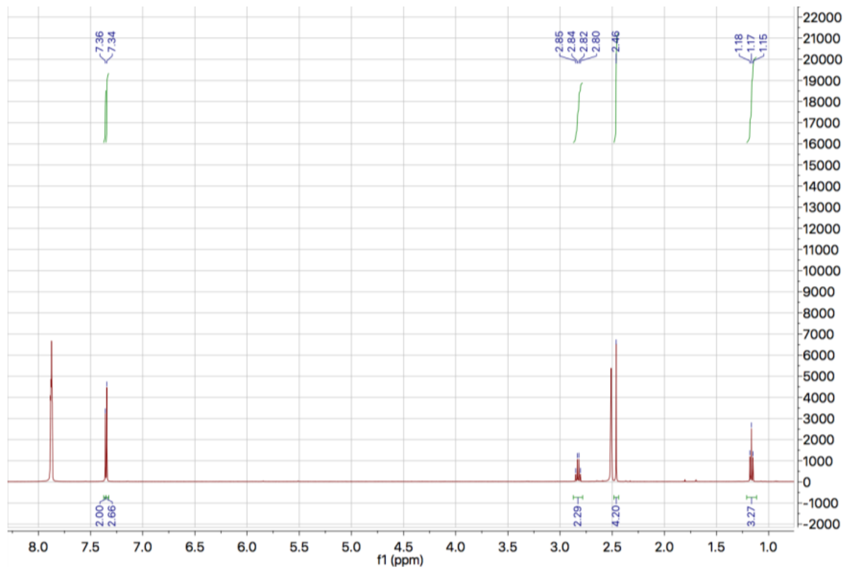

Fig. S34. ^{1}H-NMR spectrum of a washed and evacuated sample of SOD-Zn(**EtIm**)(**MeIm**) prepared by ball milling, after dissolution in DCl and DMSO-d_6. The integration of signals indicates the stoichiometric ratio **MeIm**:**EtIm** of ca. 60:40.

3.5. Solid-state nuclear magnetic resonance (ssNMR) spectroscopy

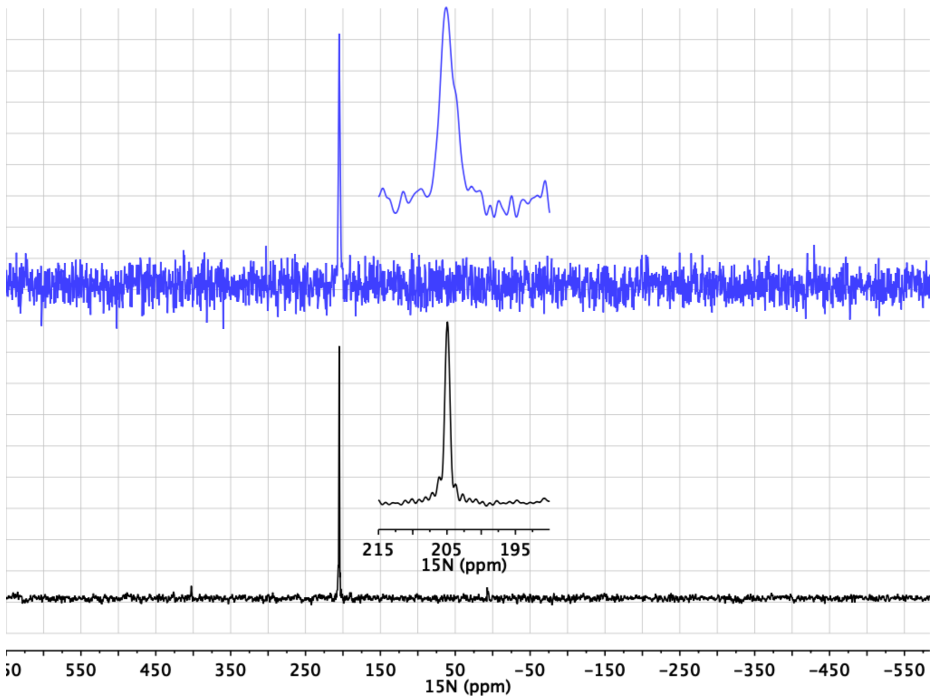

Fig. S35. Solid state ^{15}N-NMR spectrum of ZIF-8 (black) and SOD-Zn(**EtIm**)(**MeIm**) (blue) products of RAM after washing and evacuation. The ^{15}N-NMR spectrum of SOD-Zn(**EtIm**)(**MeIm**) is broader and indicated by shoulder for a different Zn-N environment of imidazoles.

3.6. N_2 sorption isotherms for surface area measurements

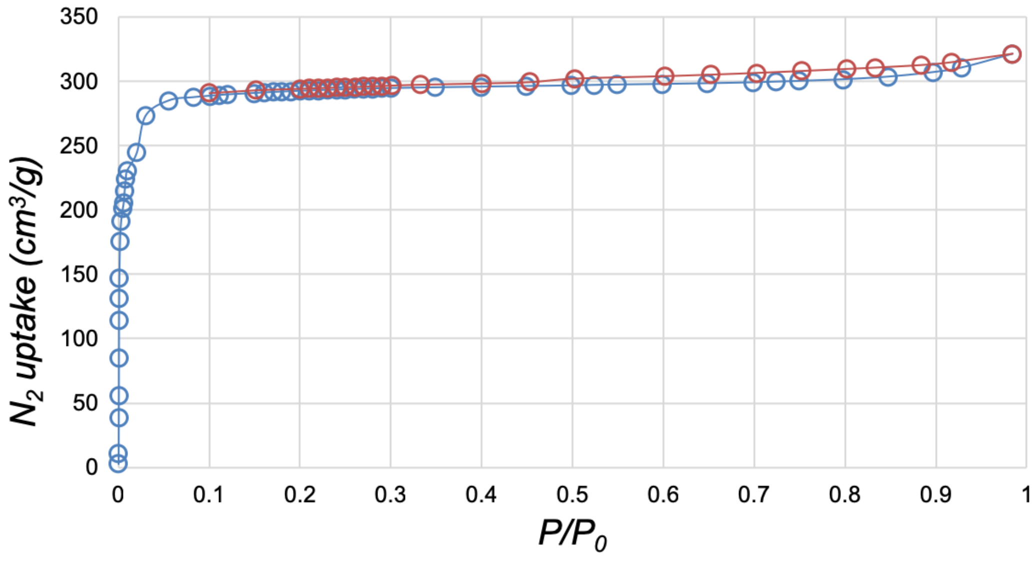

Fig. S36. Nitrogen desorption and adsorption isotherms run at 77K for ZIF-8 prepared by LA-RAM.

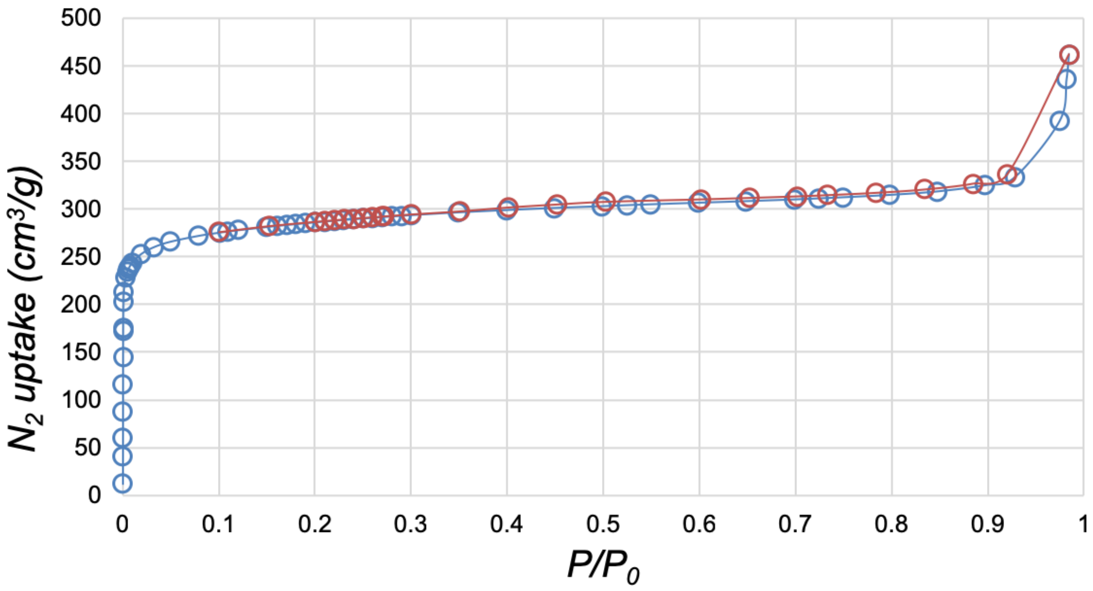

Fig. S37. Nitrogen desorption and adsorption isotherms run at 77K for SOD-Zn(**VIm**)$_2$ prepared by LA-RAM.

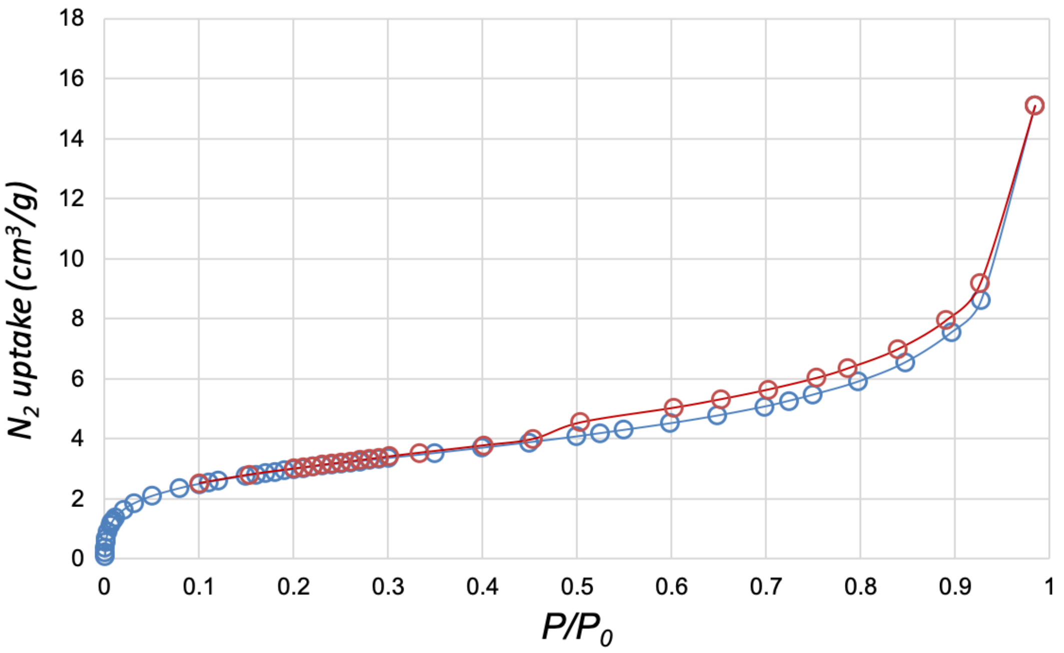

Fig. S38. Nitrogen desorption and adsorption isotherms run at 77K for ZIF-L.

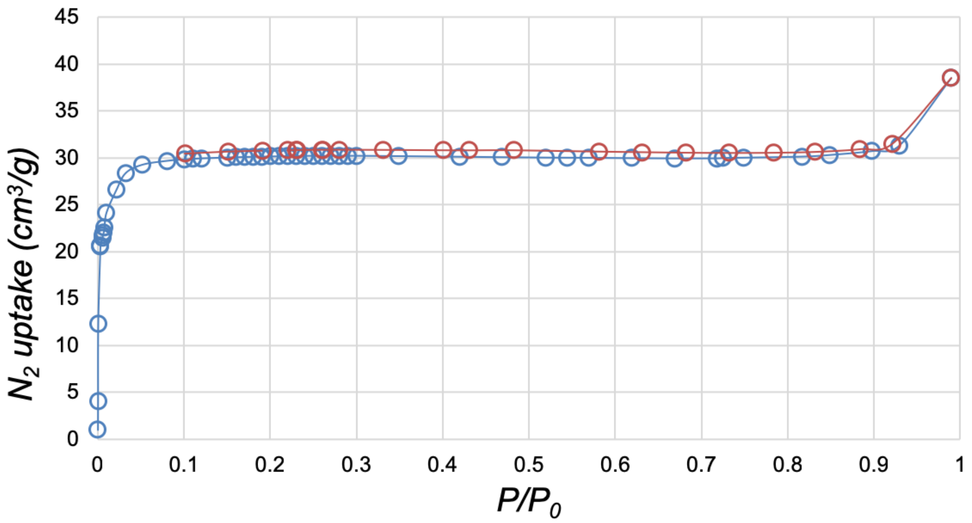

Fig. S39. Nitrogen desorption and adsorption isotherms run at 77K for Co-ZIF-L.

References:

1. Titi, H. M.; Marrett, J. M.; Dayaker, G.; Arhangelskis, M.; Mottillo, C.; Morris, A. J.; Rachiero, G. P.; Friščić, T.; Rogers, R. D. Hypergolic Zeolitic Imidazolate Frameworks (ZIFs) as Next Generation Solid Fuels: Unlocking the Latent Energetic Behavior of ZIFs. *Sci. Adv.* **2019**, *5*, No. eaav9044.
