# Supplementary material for: Simple, scalable mechanosynthesis of metal–organic frameworks using liquid-assisted resonant acoustic mixing (LA-RAM)
Source: Chem Sci. 2020 Feb 27;11(29):7578–84. doi: 10.1039/d0sc00333f (PMC8159441; doi:10.1039/d0sc00333f)
Supplement: SC-011-D0SC00333F-s002 [file SC-011-D0SC00333F-s002.pdf]

Electronic Supplementary Material (ESI) for Chemical Science.
This journal is © The Royal Society of Chemistry 2020

New Approach to Synthesising Metal-organic Frameworks

Manufacturing materials in chemistry can require laborious and environmentally toxic solvent-based processes

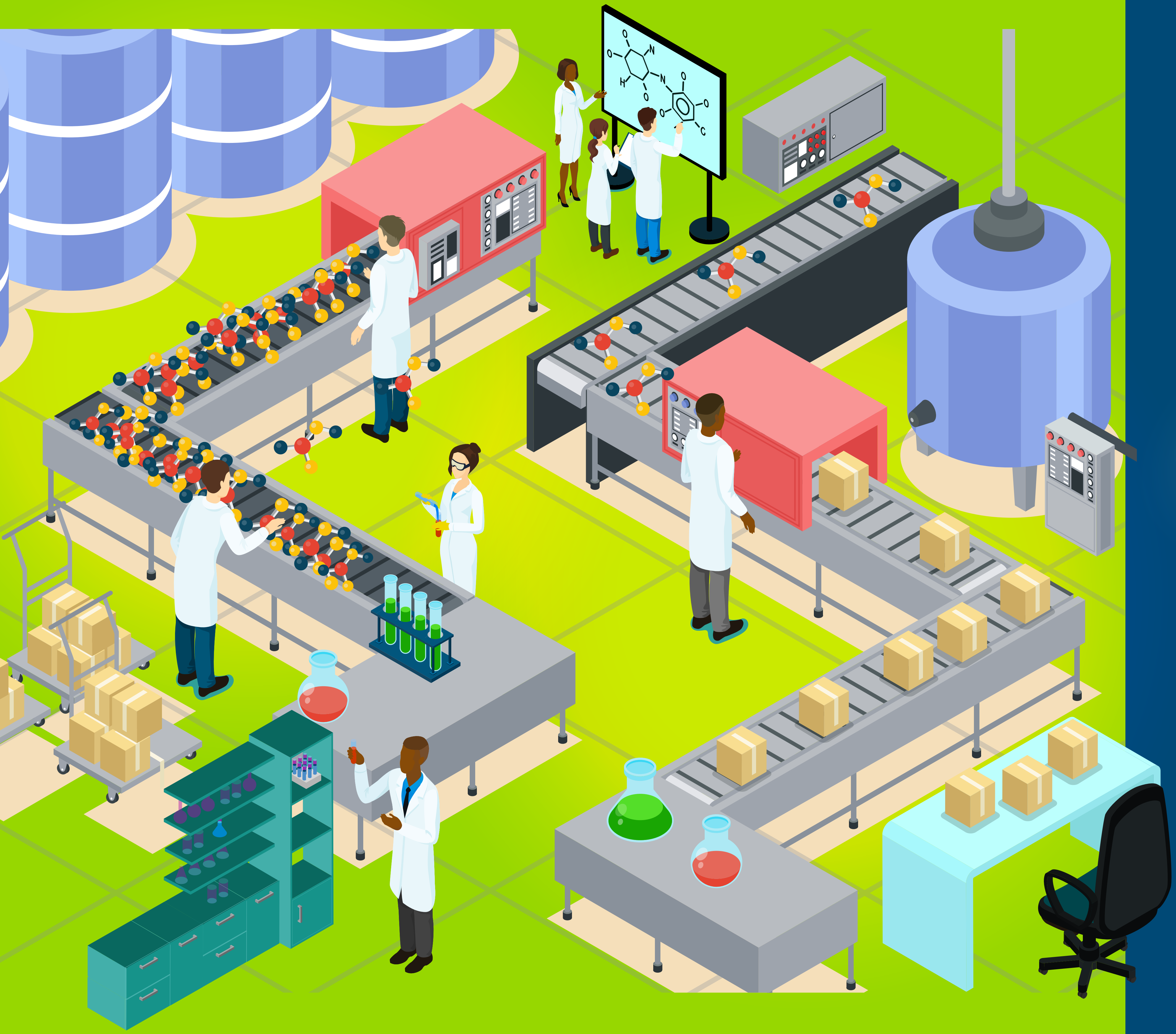

Sample agitation using ultrasonic or acoustic frequency is a solution, but this has been applied to only cocrystals until now

Can we use these methods to synthesise metal-organic frameworks?

New liquid-assisted resonant acoustic mixing (LA-RAM) approach

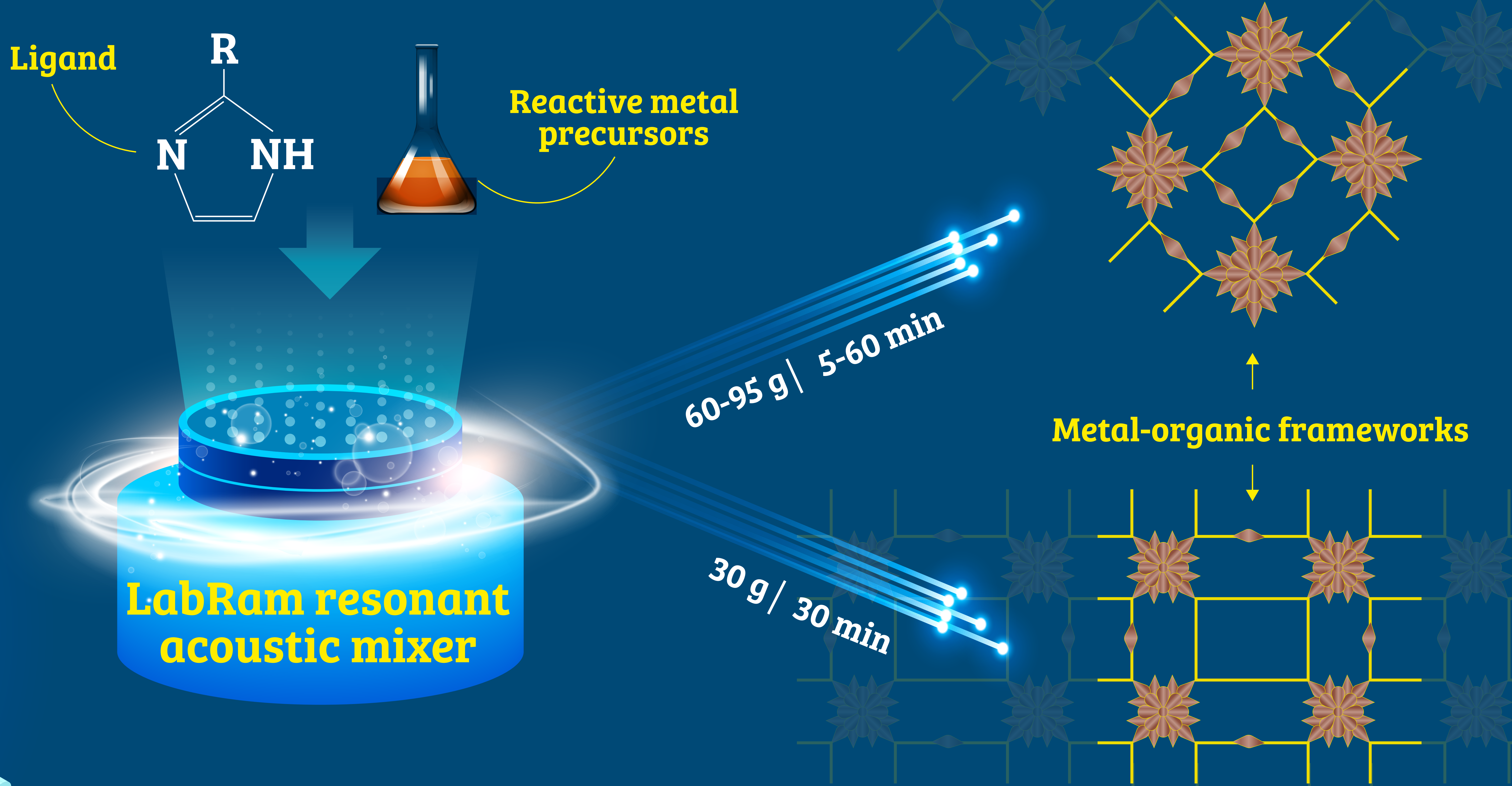

Straightforward scale-up from milligrams to at least 25 g

A greener, more economical method for the large-scale synthesis of metal-organic frameworks was developed

Chemical Science

Simple, scalable mechanosynthesis of metal organic frameworks using liquid-assisted resonant acoustic mixing (LA-RAM)

Friščić *et al.* (2020) | DOI: 10.1039/D0SC00333F

ROYAL SOCIETY OF CHEMISTRY
